# Abnormal *TP53* Predicts Risk of Progression in Patients With Barrett’s Esophagus Regardless of a Diagnosis of Dysplasia

**DOI:** 10.1053/j.gastro.2021.10.038

**Published:** 2021-10-29

**Authors:** Mark Redston, Amy Noffsinger, Anthony Kim, Fahire G. Akarca, Marianne Rara, Diane Stapleton, Laurel Nowden, Richard Lash, Adam J. Bass, Matthew D. Stachler

**Affiliations:** 1 Department of Pathology, Brigham and Women’s Hospital and Harvard Medical School, Boston, Massachusetts; 2 Inform Diagnostics, Irving, Texas; 3 Department of Molecular Oncology, Dana Farber Cancer Institute, Boston, Massachusetts; 4 Department of Pathology, University of California San Francisco, San Francisco, California; 5 R Lash MD, LLC, Southlake, Texas; 6 Eli and Edythe L. Broad Institute, Cambridge, Massachusetts

**Keywords:** Barrett’s Esophagus, Surveillance, Cancer Risk Stratification, *TP53*

## Abstract

**BACKGROUND AND AIMS:**

Barrett’s esophagus (BE) is the precursor to esophageal adenocarcinoma. A major challenge is identifying the small group with BE who will progress to advanced disease from the many who will not. Assessment of p53 status has promise as a predictive biomarker, but analytic limitations and lack of validation have precluded its use. The aim of this study was to develop a robust criteria for grading abnormal immunohistochemical (IHC) expression of p53 and to test its utility as a biomarker for progression in BE.

**METHODS:**

Criteria for abnormal IHC of p53 were developed in BE biopsies and validated with sequencing to assess *TP53* mutations. The utility of p53 IHC as a biomarker for progression of BE was tested retrospectively in 561 patients with BE with or without known progression. The findings were prospectively validated in a clinical practice setting in 1487 patients with BE.

**RESULTS:**

Abnormal p53 IHC highly correlated with *TP53* mutation status (90.6% agreement) and was strongly associated with neoplastic progression in the retrospective cohorts, regardless of histologic diagnosis (*P* < .001). In the retrospective cohort, abnormal p53 was associated with a hazard ratio of 5.03 (95% confidence interval, 3.88–6.5) and a hazard ratio of 5.27 (95% confidence interval, 3.93–7.07) for patients with exclusively nondysplastic disease before progression. In the prospective validation cohort, p53 IHC predicted progression among nondysplastic BE, indefinite for dysplasia, and low-grade dysplasia (*P* < .001).

**CONCLUSIONS:**

p53 IHC identifies patients with BE at higher risk of progression, including in patients without evidence of dysplasia. p53 IHC is inexpensive, easily integrated into routine practice, and should be considered in biopsies from all BE patients without high-grade dysplasia or cancer.

## Graphical Abstract

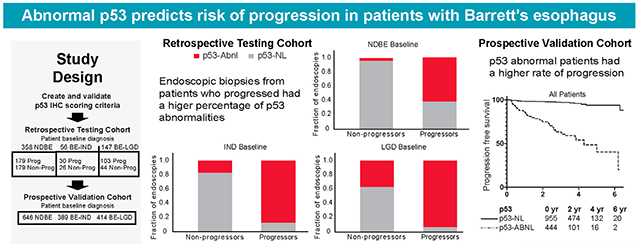

Esophageal adenocarcinoma (EAC) is an increasing cause of morbidity and mortality and health care burden, given its dismal 5-year survival rate and its striking increase in incidence.^1,2^ EAC arises from a preneoplastic precursor, Barrett’s esophagus (BE), which forms in response to reflux injury to the lower esophagus. The prevalence of BE in the US population has been estimated to be from 1.6% to 10% of adults, putting millions at heightened risk of EAC.^3,4^ Patients with BE are currently recommended to undergo frequent endoscopic surveillance, wherein biopsies are evaluated for histopathologic signs of progression, namely dysplasia. However, the annual incidence of progression among patients with BE remains low, estimated at ≤0.33%.^5^ Coupled with the large population with BE, this low risk of progression challenges the feasibility and cost-effectiveness of surveillance. An improved method of identifying which patients are at increased risk of progression, especially in the large population of those with nondysplastic BE, would greatly facilitate developing more effective surveillance and treatment strategy.

Currently, the presence of dysplasia based on pathologic assessment of BE biopsies is used to select patients for more intense surveillance or ablation/endoscopic mucosal resection. However, there are limitations to relying on dysplasia as the sole sign of increased cancer risk. First, there is substantial interobserver variability in histologic grading of dysplasia.^6–9^ Second, we lack accepted biomarkers to identify at-risk patients among the large population of patients with nondysplastic BE (NDBE). Furthermore, evidence now suggests that progression from dysplasia to cancer can occur more rapidly than originally thought,^10^ indicating that current surveillance strategies focused on finding patients in the window of time between onset of dysplasia and development of advanced cancer may be ineffective. Improved methods to identify high-risk patients with BE before the onset of dysplasia may both enhance the efficacy of screening and provide economic value by focusing resources on the minority of patients with BE who may ultimately progress.

In attempts to improve patient stratification, previous biomarker studies have queried mutations, chromosomal alterations, copy number/aneuploidy^11–14^ and methylation of specific genes.^15^ Until robust and cost-effective molecular diagnostics can be developed, there is an immediate need for biomarkers that are inexpensive, can be adopted quickly by current clinical laboratories, and can be interpreted readily by pathologists. Mutation or aberrant expression of tumor suppressor p53 has been identified as a candidate risk factor for progression in BE,^14,16–18^ but p53 IHC is currently not recommended for risk stratification.^19,20^ We sought to determine the applicability of p53 IHC to risk stratify patients with NDBE, BE indefinite for dysplasia (BE-IND), or BE with low-grade dysplasia (BE-LGD), and identify those most likely to progress to either BE with high-grade dysplasia (BE-HGD) or EAC using large, representative collections of routine screening and surveillance biopsies taken at community endoscopy centers throughout the United States.

## Methods

### Patient Selection (Retrospective Testing Cohort)

After Institutional Review Board approval, the pathology records of Inform Diagnostics (Irving, TX), a national gastrointestinal pathology laboratory providing services to gastroenterologists throughout the United States, were searched from 2001 to 2011 to identify patients who were undergoing endoscopic surveillance for BE. These patients were divided into progressors (all available patients having a baseline diagnosis of NDBE, BE-IND, or BE-LGD, followed by a diagnosis of BE-HGD or EAC) and nonprogressors (patients having a baseline diagnosis of NDBE, BE-IND, or BE-LGD with at least 3 years of follow-up without progression confirmed by at least 1 additional endoscopy with biopsies) (Supplementary Methods). Baseline endoscopies were defined as the first endoscopy with a diagnosis of BE seen at Inform Diagnostics (Supplementary Figure 1). All samples underwent blinded central pathologic review of sections cut concurrently with the IHC slides to confirm the diagnosis. All morphologic review was blinded to outcome and p53 IHC status, and all p53 IHC review was blinded to outcome and morphologic diagnosis. Clinical characteristics of patients are summarized in Table 1. This retrospective testing cohort was classified into the 3 groups (group 1: NDBE, group 2: BE-IND, and group 3: BE-LGD) based on the baseline diagnosis and was used for analysis of the baseline samples. As a patient’s diagnosis may change over time, despite their group assignment, all patients were combined and analyzed together when analysis included all available time points.

**Figure 1.**
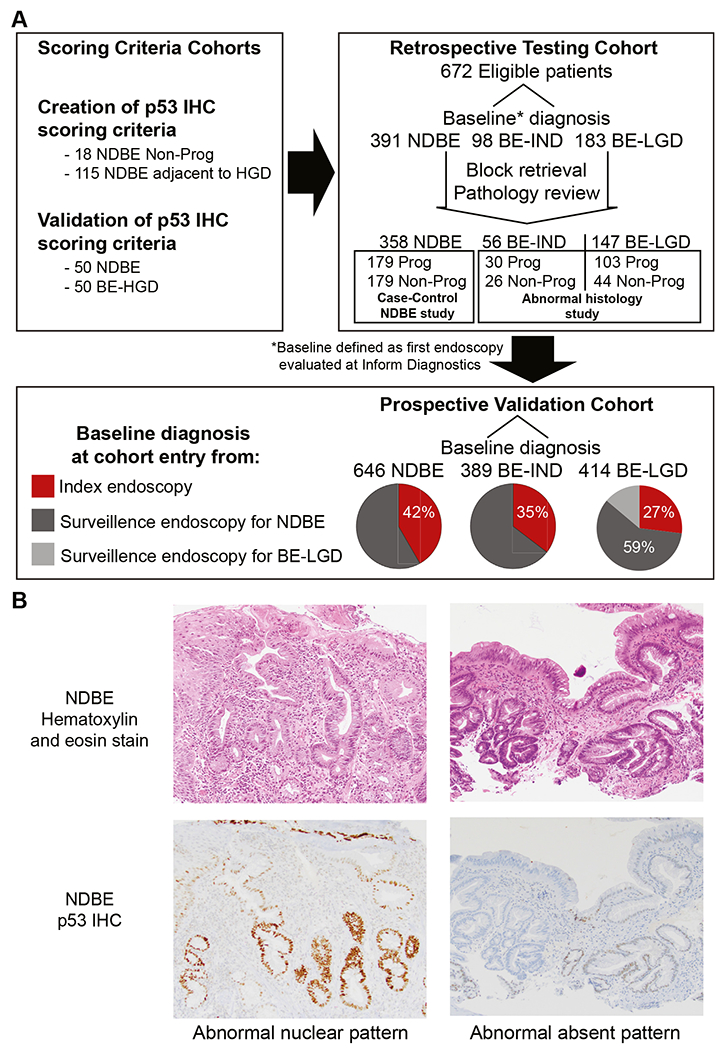
Barrett’s samples and p53 IHC staining. (*A*) Diagram of the samples used for the creation of IHC scoring criteria, testing cohorts, and validation cohorts. (*B*) Example *photomicrographs* of H&E or p53 immunohistochemistry-stained slides.

For the entire retrospective testing cohort, pathology records identified 313 potential nonprogressors and 359 potential progressors, for a total of 672 eligible study participants (Supplementary Table 2). There were 111 people (16.5%) excluded because of lack of availability of slides or tissue, or discrepant central pathology review that excluded the person from further study (Supplementary Table 3). A total of 46 participants (6.8%) were reclassified from one study group to another, predominantly because of a change in baseline diagnosis during central pathology review (Supplementary Table 4). After exclusions and reclassifications, a total of 249 nonprogressors and 312 progressors were included in the study. To look at identification of both possible incident BE-HGD or EAC and subsequent progression and to obtain a complete picture of the timing of acquisition of p53 abnormalities before progression, all available samples/time points before progression were included. As described above, the retrospective testing cohort was broken down into 3 sets to analyze the baseline endoscopies; a case-control study of patients with a baseline diagnosis of NDBE, a cohort study of patients with BE-IND, and a cohort study of patients with BE-LGD (Figure 1A). For the NDBE case-control study, controls were matched to cases based on age and sex and were required to have at least 3 years of endoscopic and biopsy follow-up confirming no progression. During the selection, priority was given to controls with the most recent follow-up. Length of BE was unknown for all patients and therefore could not be matched. To this point, the mean number of pathology blocks per endoscopy was somewhat greater in the NDBE progressors compared with the NDBE nonprogressors (1.67 vs 1.25; *P* < .001), suggesting that some NDBE progressors may have had longer-segment BE. For the BE-IND and BE-LGD cohort studies, these included all identified patients that fit the inclusion criteria and had either confirmed progression or at least 3 years of biopsy-confirmed follow-up without progression or treatment. All patients with prior endoscopic/surgical therapy were excluded from the study.

### Patient Selection (Prospective Validation Cohort)

The p53 IHC scoring criteria developed in the initial retrospective cohort were adopted by the Inform Diagnostics gastrointestinal pathology teams for standardized reporting of p53 IHC on the pathology reports and was implemented in June 2011 in routine diagnostics. p53 IHC was performed in 3 different laboratories (Massachusetts, Texas, and Arizona). A total of 41 pathologists signed out the p53 IHC results during their clinical review of cases. Patients were divided by histologic diagnosis of their baseline endoscopy, as well as whether the baseline diagnosis was the patient’s true index endoscopy or whether they were in a surveillance program. All patients with prior or subsequent endoscopic/surgical therapy were excluded from the study. To determine how p53 IHC could be implemented in practice, analyses were performed using data from the original pathologic diagnosis. However, all diagnoses of progression were reviewed to confirm the diagnosis.

### p53 Immunohistochemistry and Scoring Criteria

Immunohistochemical stains for p53 were performed using the DO-7 antibody on the BenchMark XT or BenchMark ULTRA automated slide staining systems with the OptiView or Ultra-View detection kits (Ventana Medical Systems, Tucson, AZ) according to the manufacturer’s recommendations. The percentage of nuclei with positive staining was scored on an intensity scale of 0–3, with 0+ representing no staining and 3+ representing very strong staining (Figure 1B). Abnormal (p53-ABNL) staining was considered either 2–3+ nuclear positivity in >50% of cells in at least 1 crypt base or glandular profile, or within a contiguous focus of at least 20 surface epithelial cells. Alternatively, p53-ABNL was also identified in case of total absence of staining (0+) in all epithelial cells of at least 1 crypt base or glandular profile, or strong cytoplasmic staining with complete absence of nuclear staining (rare) (Supplementary Table 1). We chose >50% 2–3+ nuclear positivity as a cutoff to define abnormal p53 expression because it had 100% specificity for an association with concurrent HGD and could be rapidly assessed by a pathologist, potentially making it a practical biomarker for routine clinical use. A detailed description of the development of the p53 IHC scoring criteria is provided in the Supplementary Methods.

### Central Pathology Review

For the retrospective testing cohorts (Figure 1A), to ensure accurate and consistent pathologic diagnoses of the specific samples used for the above analyses when tissue blocks of archival BE samples were processed to generate slides for p53 IHC, new H&E-stained slides were created and submitted for pathologic review (Supplementary Methods). Pathologists were blinded to diagnosis, outcome, and p53 status.

### Statistical Analysis

A Fisher exact test was used to compute *P* values from a 2 × 2 contingency tables. A *t* test was used to compare mean values of 2 (continuous variable) groups. For the retrospective cohort analysis including all time points, we used time-dependent covariate, Cox-proportional hazard regression to account for p53 status or diagnosis change reported in the intermediate samples within patients. We accounted for change in the p53 status and the diagnosis by including them as time-dependent (varying) covariates. Difference in the hazard of progression due to age at the index sample date was accounted for by modeling the age effect flexibly using smoothing splines. Smoothing splines are commonly used to flexibly account for the background hazard difference due to a continuous variable (like age) of which the effect is not of primary interest. Difference in the hazard due to the diagnosis was accounted for by stratifying the analysis by diagnosis. A log-rank test was used to compare Kaplan-Meier curves between p53-ABNL and p53-NL prospective validation cohorts. All *P* values were 2-tailed.

## Results

### Patient Selection and Cohort Design

The retrospective cohort clinical characteristics are summarized in Table 1. The characteristics between cases and controls were comparable. However, baseline BE-IND and BE-LGD progressors were less likely to be female than nonprogressors. There was no association detected between age at diagnosis and p53 IHC status (Supplementary Table 5). We included cases who may have had incident progression (defined as NDBE, BE-IND, and BE-LGD samples diagnosed within 1 year of a BE-HGD or EAC diagnosis), as even with modern endoscopic techniques, focal HGD and early EAC may be missed during endoscopic screening. Patients with prior endoscopic/surgical therapy were excluded from the study. After exclusions (see Supplementary Tables 2–4), there were 179 patients with available NDBE samples taken before subsequent progression to HGD or EAC and 179 nonprogression patients with NDBE who were matched on patient age and sex. In addition, 56 patients with a baseline diagnosis of BE-IND (30 progressors and 26 nonprogressors) and 147 patients with a baseline diagnosis of BE-LGD (103 progressors and 44 nonprogressors) were identified and studied as patients with abnormal baseline histology.

There were 1438 patients included in the prospective validation cohort. Six hundred and forty-six patients had a baseline NDBE diagnosis where 270 of the baseline NDBE endoscopies were a true index endoscopy and 376 were surveillance for prior diagnosis of NDBE. Three hundred and eighty-nine patients had a baseline BE-IND diagnosis where 138 of the baseline BE-IND endoscopies were an index endoscopy and 251 were surveillance for NDBE. Four hundred and fourteen patients had a baseline BE-LGD diagnosis where 110 of the baseline BE-LGD endoscopies were an index endoscopy, 239 were surveillance for NDBE or BE-IND, and 65 were surveillance for BE-LGD.

### *Development of p53 Immunohistochemistry Scoring Criteria and Correlation With* TP53 *Mutations Status*

We first developed our p53 IHC scoring by performing IHC staining on 18 NDBE biopsies from patients with no known dysplasia and in 115 NDBE biopsies from patients with concurrent HGD, as the latter cases were most likely to have p53 abnormalities. Scoring criteria (Supplementary Methods and Table 1) were selected to show 100% specificity (ie, 0 of 18 NDBE from patients without dysplasia were positive), which yielded 39 of 115 positive NDBE biopsies (34%) in patients with concurrent HGD. The staining criteria was validated using 50 unselected NDBE biopsies and 50 BE-HGD biopsies. Abnormal p53 staining was seen in 2 of 50 of the NDBE biopsies (4%) and 48 of 50 BE-HGD biopsies (96%), confirming the scoring criteria are both sufficiently sensitive and specific.

To evaluate how p53 IHC status relates to *TP53* mutation status, we next performed *TP53* sequencing on a subset of samples. We were able to obtain adequate DNA for sequencing from 92 BE samples derived from 28 progression patients and 6 nonprogression patients. *TP53* mutations were identified in the DNA from 50 of these samples, specifically from 21 progression patients and 3 nonprogression patients. In 83 of 92 samples (90.2%), the mutational status correlated with the p53 IHC results. There were 2 samples in which a *TP53* mutation was called, but IHC was read as normal and there were 7 samples that were negative for a mutation, but read as abnormal with IHC. Interestingly, of the 7 samples negative for mutation and abnormal by IHC, 6 had an absent IHC staining pattern, including 5 samples from a single patient, raising the hypothesis of an alternative pathway of p53 silencing in that patient. In samples with both abnormal p53 IHC and a *TP53* mutation, all 10 samples with an absent p53 IHC pattern had a mutation that would likely lead to a truncated protein, confirming the importance of recognizing the absent pattern as a marker for these pathogenic *TP53* mutations. All but 1 sample with strong nuclear p53 IHC staining had a missense mutation seen recurrently in cancer. In total, p53 IHC was 96% sensitive and 83.3% specific for identifying *TP53* mutations in the sequenced samples.

### Baseline p53 Immunohistochemistry Status Predicts Progression Regardless of Histologic Diagnosis

We performed p53 IHC on the baseline endoscopies of all of the patients in the retrospective testing cohorts (NDBE, BE-IND, and BE-LGD). p53 IHC grading was performed blinded to diagnosis and progression status. Biopsies from progressors were much more likely to be p53-ABNL than biopsies from nonprogressors. In patients who progressed to advanced disease, p53-ABNL in baseline endoscopies (Supplementary Figure 1) was 89 of 179 (49.7%), 27 of 30 (90.0%), and 97 of 103 (94.2%) in NDBE, BE-IND, and BE-LGD, respectively. These numbers were dramatically lower in nonprogressing patients, with 3 of 179 (1.7%), 4 of 26 (15.4%), and 20 of 44 (45.4%) positive in NDBE, BE-IND, and BE-LGD, respectively (*P* < .00001 for all) (Supplementary Tables 6 and 7).

In our NDBE case-control testing cohort, p53-ABNL in the baseline endoscopy had a sensitivity of 50.8% and specificity of 98.3% for progression, with an odds ratio (OR) of 58 (95% confidence interval [CI], 17.9–188.5; *P* < .0001). In our abnormal histology testing cohort, p53-ABNL in the baseline endoscopy with a diagnosis of IND was associated with a sensitivity of 90.0% and specificity of 84.6% for progression with an OR of 49.5 (95% CI, 10.0–245.0; *P* < .0001). p53-ABNL in the baseline endoscopy with a diagnosis of LGD was associated with sensitivity of 94.2% and specificity of 54.6% for progression with an OR of 17.8 (95% CI, 6.4–49.5; *P* < .0001). If the control group was restricted to only patients who had 2 or more endoscopies with confirmed LGD, the results held with a slightly increased specificity of 66.7%. The results were similar when analyses were preformed using the original pathologic diagnosis (rather than the central pathology review) (Supplementary Tables 8 and 9).

### p53 Abnormal Barrett’s Esophagus Biopsies Are Highly Enriched in Patients Who Will Progress

**Figure 2.**
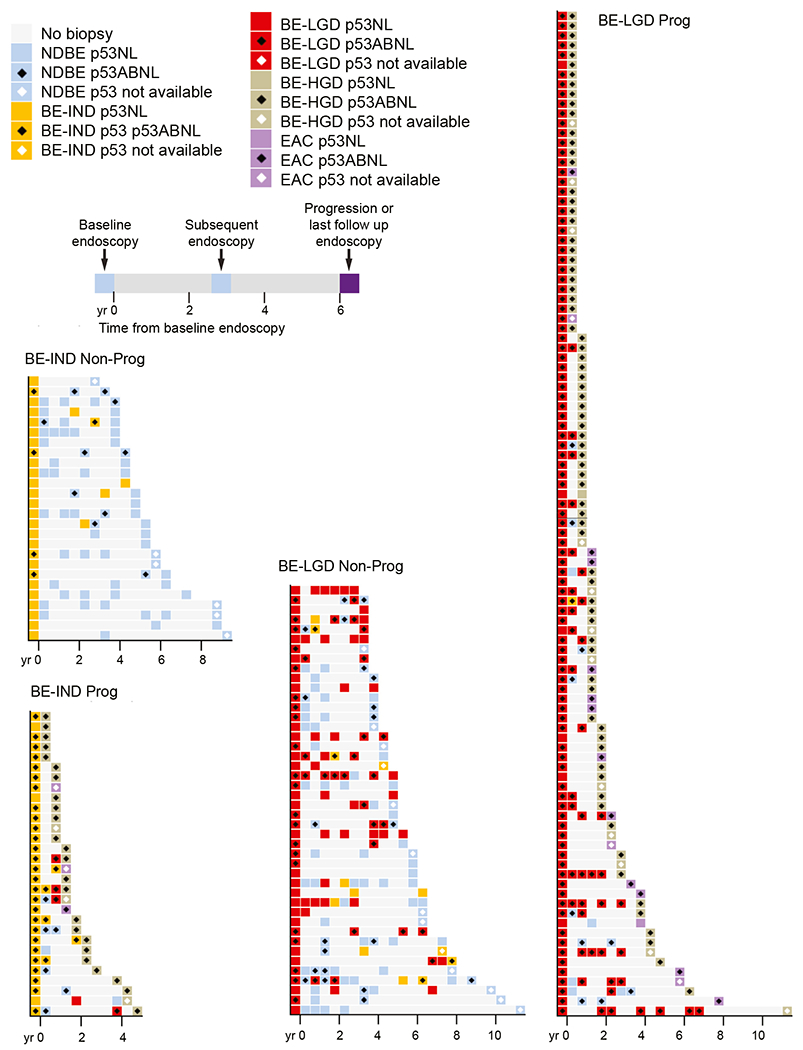
p53 IHC positivity in BE-IND and BE-LGD retrospective testing cohort. Charts showing timing, histologic diagnosis, and p53 IHC positivity for all patients with a baseline of BE-IND or BE-LGD diagnosis separated by progression status. Each *row* represents a single patient and timeline on *x-axis* shows when endoscopies occurred with baseline endoscopy at time 0.

**Figure 3.**
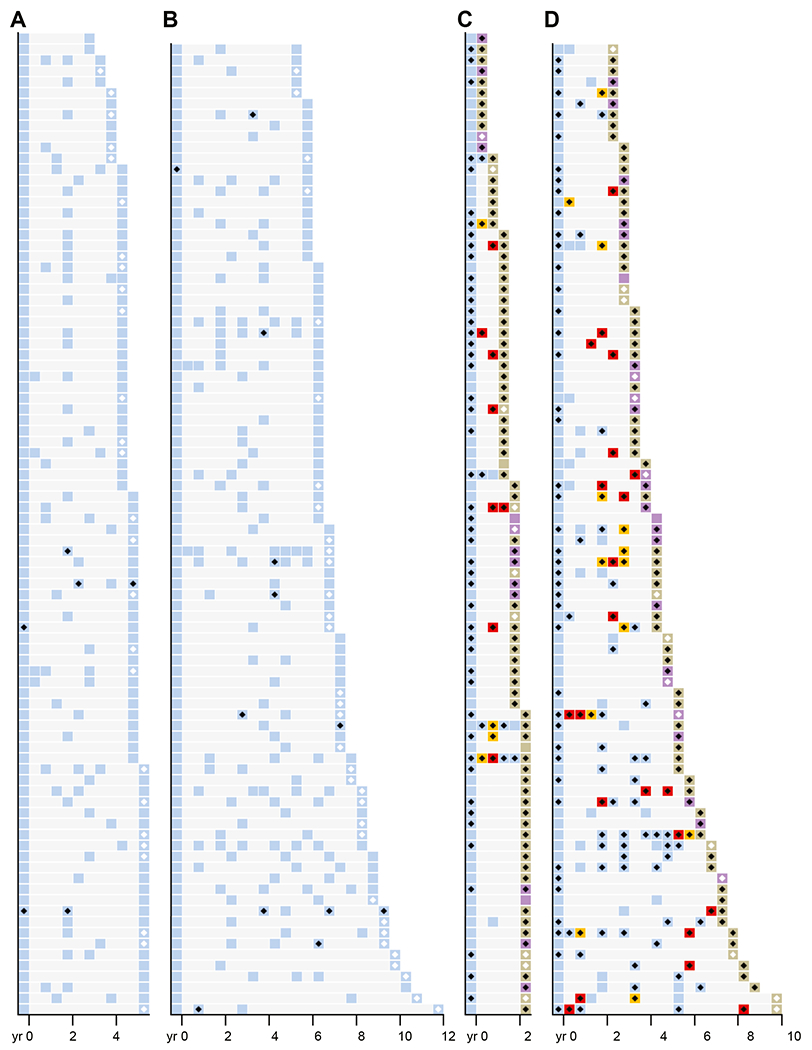
p53 IHC positivity in NDBE retrospective testing cohort. *Charts* showing timing, histologic diagnosis, and p53 IHC positivity for all patients with a baseline diagnosis of NDBE separated by progression status. Each *row* represents a single patient and timeline on *x-axis* shows when endoscopies occurred with baseline endoscopy at time 0. (*A*) Nonprogression patients with less than 6 years of follow-up. (*B*) Nonprogression patients with more than 6 years follow-up. (*C*) Progression patients with less than 3 years before progression. (*D*) Progression patients with more than 3 years of follow-up.

**Figure 4.**
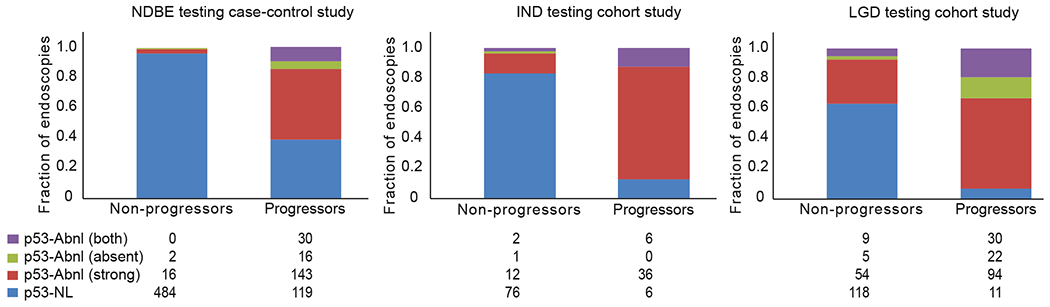
Summation and breakdown of p53 IHC by type of staining pattern. *Bar graphs* show the fraction of each staining pattern (normal, abnormal strong nuclear, abnormal absent, or abnormal both strong nuclear and absent) in the retrospective testing cohort based on baseline endoscopy diagnosis. The *numbers* below represent the total number of endoscopies for each category within the *column* above.

Figures 2 and 3 show accession timeline charts of diagnoses and p53 IHC in each endoscopy over time for all nonprogressors and progressors, grouped by baseline histology, and sorted by duration of follow-up. In total, across all of the retrospective cohort, 381 of 519 endoscopies from progressors were p53-ABNL and 101 of 780 endoscopies from non-progressors were p53-ABNL. Figure 4 shows the fraction of samples with the different categories of p53-ABNL (strong nuclear or absent). In total, for the retrospective cohorts 25%, 14%, and 36% of NDBE, IND, and LGD p53-ABNL endoscopies from progressors, respectively, contained the absent staining pattern. For the nonprogressors the percentage of p53-ABNL endoscopies that contained the absent pattern were 11%, 20%, and 20% for NDBE, IND, and LGD patients, respectively. Although these percentages included patient with both increased and absent staining, there were 9 (8%), 0, and 17 (17%) NDBE, IND, and LGD progression patients, respectively, with exclusively absent staining pattern in all of their p53 abnormal endoscopies.

As many patients had samples from multiple surveillance time points taken after the baseline endoscopy but before progression and the subsequent endoscopies could have a different histologic diagnosis compared with baseline, we used time-dependent covariate Cox-proportional hazard regression to analyze p53 IHC across all samples in the retrospective testing cohort. p53-ABNL corresponded to a hazard ratio (HR) of 5.025 (95% CI, 3.879–6.506; *P* < .0001) for the combined cohort. This controlled for age, sex, and histologic diagnosis.

We then limited our analysis to only patients who exclusively had a NDBE diagnosis throughout surveillance and who had biopsies more than 1 year before progression to determine the rate of p53 positivity in this most restricted population. In this group of clinically and histologically very-low-risk patients, p53 IHC was abnormal in 90 of 179 (50.3%) endoscopies from 72 of 127 patients who progressed and only 18 of 500 (3.6%) endoscopies from 14 of 179 patients who did not progress across all preprogression time points. This corresponded to a hazard ratio of 5.274 (95% CI, 3.934–7.072; *P* < .0001) for p53-ABNL in this exclusively NDBE patient subset. Interestingly, 8 of the 14 nonprogression patients with p53-ABNL endoscopies were only p53-ABNL in the latter part of their follow-up, with 3 years or less subsequent follow-up since becoming p53-positive (Figure 3).

### p53 Immunohistochemistry Allows Identification of Higher-Risk Patients Earlier and More Frequently Than a Diagnosis of Low-Grade Dysplasia or Indefinite for Dysplasia

**Figure 5.**
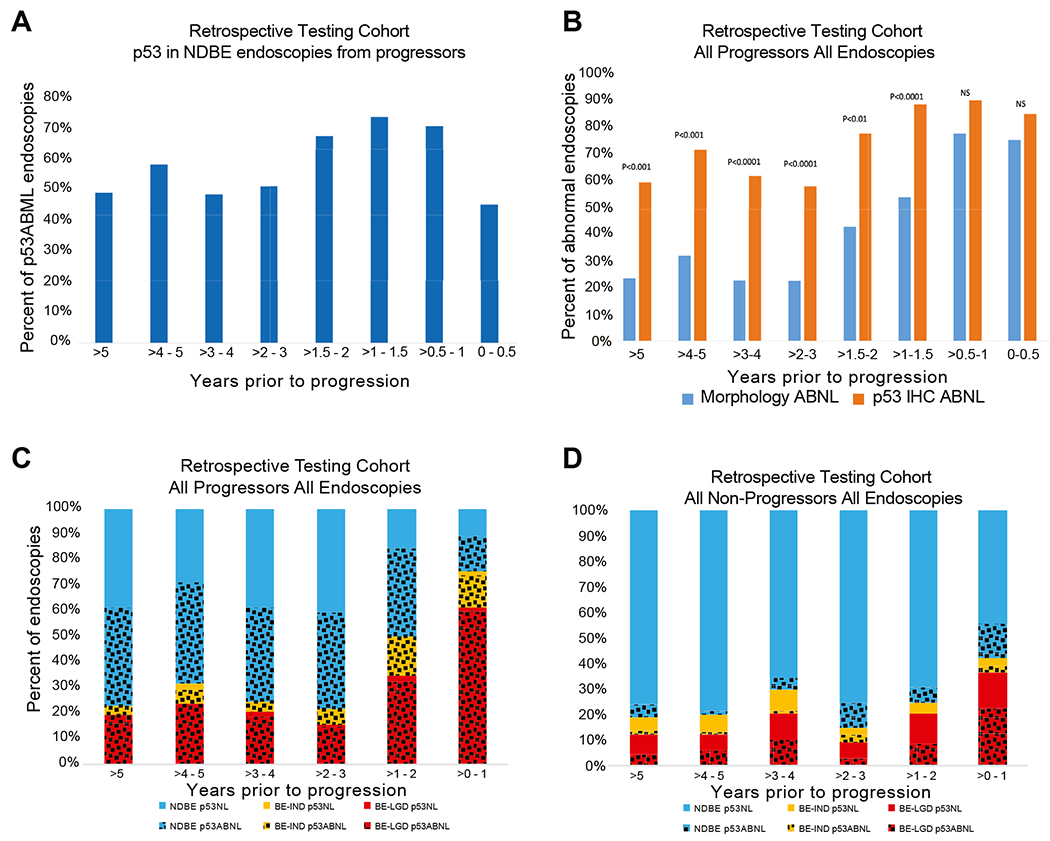
p53-ABNL vs time to progression. (*A*) Percent of endoscopies from patients in the baseline NDBE cohort that were p53-ABNL taken at different time points before progression. (*B*) Comparison of the percentage of endoscopies positive for either a histologic abnormality (BE-IND or BE-LGD) or p53 IHC broken down over different time periods before progression. (*C, D*) Frequency of first morphologic or first p53 abnormality in all progressors (*C*) or nonprogressors (*D*). At every point in time, >50% of progressors have had at least 1 p53-ABNL biopsy, and substantially <50% have had a morphologic abnormality until the final preprogression year.

As the current standard is to use histologic abnormalities (ie, dysplasia) to define high-risk features, we evaluated the preprogression biopsies in all patients with subsequent progression to determine when p53-ABNL emerged relative to the detection of a histologic abnormality. The prevalence of p53-ABNL was calculated for sequential time points before progression and was found to be stable over time (Figure 5 and Supplementary Figure 2). The only notable drop of p53-ABNL occurred in the small subgroup of NDBE biopsies taken more than 7 years before progression (5 of 18 [27.8%] p53-ABNL; χ^2^ NS compared with NDBE biopsies within 7 years of progression 161 of 315 [51.0%]). In contrast, a diagnosis of BE-IND or BE-LGD occurred closer to progression with the prevalence of histologic abnormalities (BE-IND or BE-LGD) in all progressor biopsies falling steadily between 1 and 3 years before progression (Figure 5). Abnormalities in p53 IHC were present at a higher frequency than abnormalities in morphologic diagnosis at all time points, and this difference was most striking at time points more than 2 years before progression. Importantly, at 3 to 5 years before progression, which encompasses the current surveillance guideline of patients with BE with no prior dysplasia, morphologic abnormalities were found in only 23 of 87 (26.4%) endoscopies in patients with subsequent progression. In contrast, p53 abnormalities were present in 57 of 87 (65.5%) of these endoscopies (χ^2^, *P* < .00001 for both) (Figure 5C and D). Overall, p53-ABNL was much more prevalent in preprogression biopsies, occurring more frequently and earlier, than any morphologic abnormality (BE-IND and BE-LGD combined).

### P53 Immunohistochemistry as Biomarker for Progression

As examples where a biomarker in NDBE samples would have been highly valuable clinically, in our study set there were 32 patients with NDBE who progressed to fully invasive esophageal adenocarcinoma while on surveillance. Histologic abnormalities were identified before invasive carcinoma progression in only 6 of 32 (18.8%) (Supplementary Figure 3). Among the remaining 26 patients, histology failed to alert the clinicians that these patients were at high risk of developing cancer. However, abnormal p53 IHC was present in all 6 patients with histologic abnormalities, and in an additional 11 of 26 patients (42.3%) who only had NDBE diagnosed before developing invasive EAC. Overall, p53 IHC was significantly more sensitive than histology at identifying an abnormality before the development of invasive EAC (6 of 32 vs 17 of 32; Fisher exact test, *P* < .01). Among these 32 cases, there were 24 patients who progressed from NDBE to invasive cancer before their next recommended endoscopy (interval progression). Regardless of whether a higher-grade lesion was not biopsied in the prior screening endoscopy or these patients progressed rapidly, a biomarker warning the clinicians that these patients were at heightened risk and should be followed more closely would likely have enabled earlier diagnosis or prevention. In these 24 patients, 11 (46%) were p53-ABNL.

### p53 Immunohistochemistry Stratifies Patients in a Routine Practice Setting

**Figure 6.**
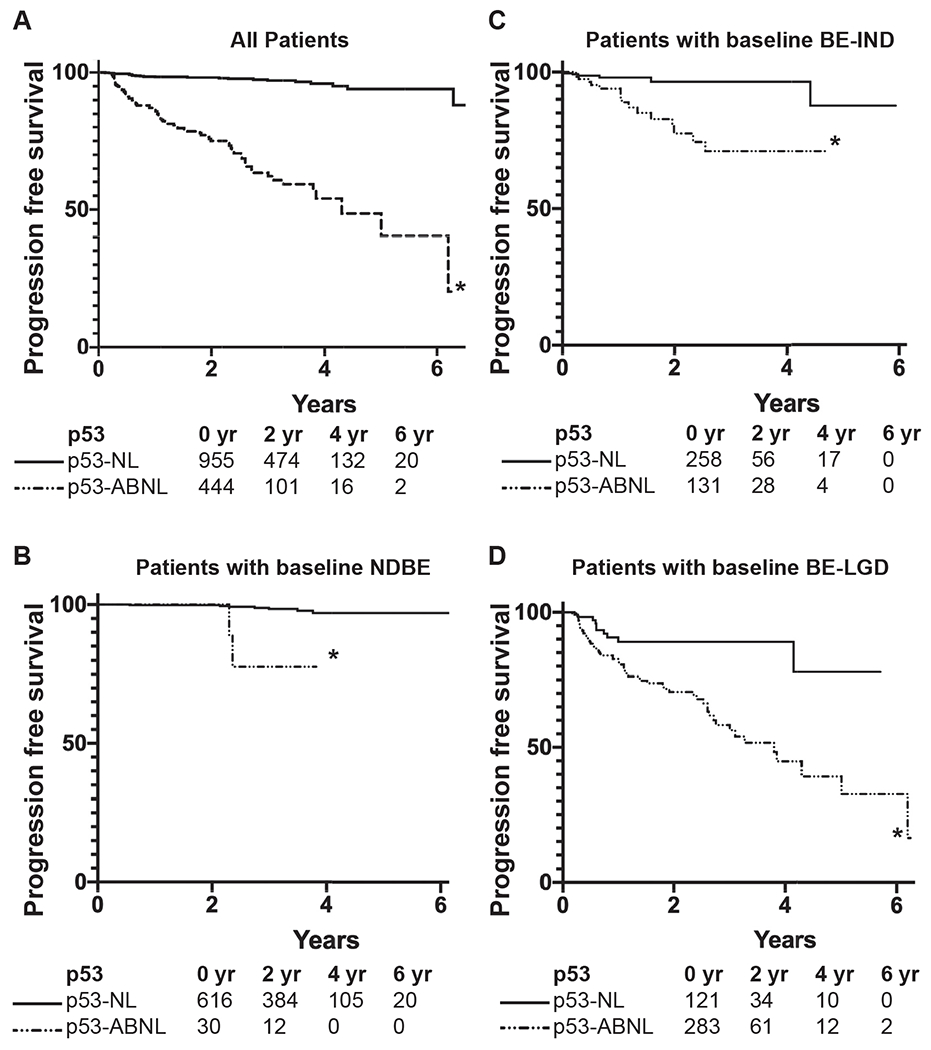
p53 IHC patient stratification in a prospective validation cohort. Kaplan-Meier curves for progression to BE-HGD/EAC-free survival in the entire prospective cohort (*A*) and patients with baseline diagnosis of NDBE (*B*), BE-IND (*C*), and BE-LGD (*D*). All patient numbers and statistical analysis are reported in Supplementary Table 10. *P < .05 p53-NL vs p53-ABNL.

To validate our findings in the retrospective cohort, we next wanted to determine whether the addition of p53 IHC to standard histologic diagnosis would improve Barrett’s outcome prediction in a routine clinical setting. Prospective p53 IHC, using the same scoring criteria described above, was adopted for routine signout of clinical BE biopsies at Inform Diagnostics in June 2011. Pathology records were searched to identify all BE patients with a reported p53 IHC result and a subsequent follow-up biopsy. In total, 1449 patients were included. Of 389 cases of BE-IND and 414 cases of BE-LGD identified with p53 IHC, 22 (5.6%) and 78 (20%) progressed to either HGD or EAC, respectively. Among 646 NDBE cases, in the limited follow-up time, 20 (3.1%) progressed to LGD and 10 (1.5%) progressed to HGD/EAC. Kaplan-Meier analysis (Figure 6) showed the ability of p53 IHC to clearly stratify patients in all cases (log-rank test, *z* = 3.81, *P* < .001 for BE-IND; *z* = 3.76, *P* < 0.001 for BE-LGD; *z* = 9.81, *P* < .001 for NDBE to LGD; and *z* = 3.08, *P* = .002 for NDBE to HGD). In the entire cohort, p53-ABNL corresponded to an HR of 12.51 (95% CI, 7.984–19.61; *P* < .0001). Performing a subanalysis based on baseline diagnosis showed an HR of 3.29 (95% CI, 2.05–5.29; *P* = .0002) for patients with baseline BE-LGD and an HR of 5.10 (95% CI, 2.11–12.28; *P* < .0001) for patients with baseline BE-IND. Although few patients with a baseline diagnosis of NDBE progressed in the limited follow-up time, p53-ABNL suggested an HR of 11.83, although with an extremely wide 95% CI of 0.15–919.5 (*P* < .0001). In total, p53 IHC was able to stratify patients with respect to progression regardless of the histologic diagnosis in a large prospective cohort in a standard clinical setting.

To determine whether the timing of the biopsy in relation to the patient’s surveillance history may affect the results, we separated the results into the patients with p53 IHC information on their first index endoscopy (first ever diagnosis of BE), patients where the first p53 IHC was on a surveillance endoscopy for NDBE (no known BE-IND or BE-LGD), or patients with BE-LGD when the first p53 IHC was on a surveillance endoscopy after a diagnosis of BE-LGD. Kaplan-Meier curves for progression-free survival were similar for patients with an index sample or patients with only surveillance endoscopies in NDBE and BE-IND. For BE-LGD, patients identified in an index endoscopy did slightly worse than those undergoing surveillance for NDBE (log-rank test; *z* = 2.94, *P* = .014) (Supplementary Figure 4). Under all conditions of having the index endoscopy or only surveillance endoscopies and under all histologic diagnoses, p53 IHC was able to stratify progressors from non-progressors, confirming the results seen in the retrospective testing cohorts (Supplementary Figure 5 and Supplementary Table 10).

## Discussion

EAC and its associated precursor, BE, is a growing clinical problem with significant issues with both over- and underdiagnosis. There is a clear need for improved methods and biomarkers for risk stratification of patients with BE. Many studies have focused on the different molecular alterations present in BE, and the best risk stratification will likely come from a combination of molecular biomarkers.^11,15,21^ However, several barriers still exist before widespread clinical adaptation of such testing can be accomplished. Here we present data to show that an inexpensive, readily available biomarker could have immediate clinical utility. Our data greatly expand and complement previous studies examining p53 and BE. In one of the most cited studies, Kastelein et al^18^ looked at p53 IHC for risk prediction and used grading criteria similar to ours. In their well-controlled, case-control study, they had 15 patients with NDBE and 34 patients with LGD who progressed to either HGD or EAC and, similar to our study, found that 32.4% and 70.7% of biopsies from NDBE and LGD progression patients, respectively, had abnormal p53 IHC. Importantly, this study used a strict research protocol that included 4-quadrant biopsies every 2 cm of BE and performed all p53 IHC staining as a single batch. While these strict criteria are important for initial studies, for a biomarker to be clinically useful it needs to function under more “real-world” settings that often fail to meet these high standards.^22^ Our study used samples taken in the community setting under routine care and with a substantially higher number of progression patients. In the prospective validation cohort, this included IHC staining in real-time from 3 different laboratories and interpretation from 41 different pathologists. In light of past studies, we feel that obtaining our results under these real-world conditions strongly supports the robustness and reproducibility of using p53 IHC.

However, this type of study also comes with several important limitations. First, although patients with endoscopic mass lesions or therapy were excluded, information on other clinical factors, such as length of BE, was unknown. The fact that the mean number of pathology blocks per endoscopy in the retrospective testing cohort tended to be greater in the progression cohorts compared with the nonprogression cohorts—1.67 vs 1.25, 1.58 vs 1.82, and 2.84 vs 1.92 for NDBE, BE-IND, and BE-LGD, respectively (Table 1)—may indicate a higher proportion of long-segment BE or more thorough sampling among progressors. Second, given the real-world aspect of this sample set, the time between endoscopies was not consistent, and in the retrospective cohort, the time between the baseline endoscopy and first follow-up endoscopy was shorter in the patients who progressed vs those who did not. In the prospective cohorts, although the majority, not every biopsy was tested, and this selection could also bias the results. Finally, although our grading criteria developed on independent cohorts provides good stratification and correlates well with mutation status, it may be more complicated to learn and apply in routine clinical practice compared with some previous studies methods. Our study graded p53 IHC staining intensity on a 0–3+ scale with 2–3+ nuclear positivity in >50% of cells in at least 1 crypt base or glandular profile, or within a contiguous focus of at least 20 surface epithelial cells as abnormal “increased” staining and total absence of staining in all epithelial cells of at least 1 crypt base or glandular profile considered abnormal “absent” staining. These criteria require the pathologist to distinguish 0–1+ from 2–3+ staining and to recognize foci of complete absent staining (Supplementary Figure 6 and Supplementary Figure 7). Although potentially challenging, given the increased focus on HER2 grading in a somewhat similar manner, and the fact that our prospective cohort used 41 different pathologists who only received a short training, we believe that this approach can be implemented by most pathologists.

In the setting where risk of progression is low, such as for patients with NDBE, a useful biomarker will require a high specificity to reduce the number of false positives inherent to this situation. In our cohort of patients with only NDBE before progression, p53-IHC was quite specific. The sensitivity was lower but still identified half of all progressors in a cohort of patients who showed no histologic abnormalities (ie, no BE-IND or BE-LGD) before progression. If one assumes a general NDBE progression rate of 0.3% per year, our data suggest an approximately 30% risk of progression within 5 years for patients with p53-ABNL NDBE. Although the estimates for progression risk in LGD are highly variable, this calculated rate for risk of progression p53-ABNL NDBE is comparable with rates of progression in those diagnosed with BE-LGD. Given this, changing practice to treat p53-ABNL NDBE as an equivalent to how we now treat BE-LGD with yearly endoscopies would seem appropriate. However, with the low risk of progression of NDBE patients, even with a specificity in the 90s (98.3% for baseline NDBE) more false positives than true positives will occur. This poses a risk of overtreatment and increasing the overall health care burden. Interestingly, although the data in our study were somewhat limited, it appeared that when NDBE nonprogressors had a p53-ABNL result, it was more frequently followed by a p53-NL endoscopy, where NDBE progressors were more likely to have back to back p53-ABNL results (Figure 3). This may limit the number of excess endoscopies in nonprogressors if results over time demonstrate that having p53-NL samples after an abnormal result indeed denotes less need for more intense surveillance.

Given our data, and assuming no visible lesions, we suggest that a p53-ABNL NDBE result could be confirmed by repeat surveillance endoscopy at an interval of 12 months. If this endoscopy is also p53-ABNL NDBE, the patient would continue yearly surveillance until they either progress or get a p53-NL result. This will require a second endoscopy, but would limit the number of nonprogression patients being categorized as high risk to those who recurrently are p53-ABNL. If the second endoscopy is p53-NL NDBE, we would suggest no change from current recommendations. After consecutive p53-NL NDBE results, the patient is likely at very low risk of progression and can have repeat surveillance at 5 years (as opposed to current 3–5 years), as summarized in Supplementary Figure 8.

Using the decision pathway in Supplementary Figure 8, when a p53-ABNL NDBE sample would lead to a follow-up surveillance endoscopy in 1 year, there were 99 patients with NDBE progression who would have been classified as high risk based on their p53-ABNL NDBE endoscopies leading up to their progression. Of the other progression patients, 10 would have been classified as low risk, with the remaining NDBE progression patients not having had any change in care. For the NDBE nonprogressors, 16 patients would have no change in care (only 1 p53-NL and no p53-ABNL endoscopy), 155 patients would be classified as low risk after having 2 consecutive endoscopies that were p53-NL, and 14 patients would be classified eventually as high risk after having an endoscopy with a p53-ABNL result. Of the 14 patients classified as high risk, 9 of 14 had their subsequent surveillance endoscopy that was p53-NL, returning them to the normal surveillance schedule.

Currently, many patients with BE-LGD are undergoing treatment to eradicate their BE. Although potentially helpful for those with BE-LGD that will progress to cancer, studies suggest that most of these patients would not progress if left untreated.^23–26^ With endoscopic treatment for BE-LGD becoming standard, for a biomarker to be useful it needs to be highly sensitive to not miss patients when treatment would be beneficial, but allow the identification of some lower-risk patients when alternative approaches besides treatment may be better suited. In patients with BE-LGD, p53 IHC identified those who would progress with a high sensitivity. Although a proportion of the nonprogression patients were also positive, p53 IHC provided increased stratification and may reassure clinicians and patients that their decision for continued surveillance or therapy is sound. Patients with p53-ABNL BE-LGD are at increased risk of progression and a recommendation of endoscopic therapy would seem appropriate. Although p53-NL BE-LGD is at decreased risk of progression compared with p53-ABNL BE-LGD, it appears to be at increased risk compared with p53-NL NDBE. Therefore, p53-NL BE-LGD with no visible lesions could be offered a repeat endoscopy at 1 year (Supplementary Figure 8). This approach would spare nonprogressing p53-NL BE-LGD the potential morbidity associated with endoscopic treatment, but increase the chances of detecting any progressors by suggesting repeat endoscopy at a 1-year interval. Future studies will be needed to determine whether persistent p53-NL BE-LGD can have an increased surveillance interval.

The diagnosis of BE-IND is also challenging to manage and is considered to be a mixture of patients with NDBE that have developed intense reactive changes and patients who have a true dysplasia. In our prospective cohort, the p53-ABNL BE-IND patients progressed similarly to patients with a BE-LGD diagnosis. As both progress at nearly equal rates, clinically it would not be unreasonable to treat patients with p53-ABNL BE-IND in a manner similar to those with BE-LGD. The patients with p53-NL BE-IND had a significantly lower progression rate, with only 7 of 263 progressing through the duration of the study. Given this low rate of progression, these patients could likely undergo surveillance less frequently.

The potential utility of p53 IHC as a biomarker of Barrett’s progression risk has been an area of active study. Although these studies showed promise, many of them had limitations that prevented widespread adoption.^16,18,27,28^ Our study removes some of these barriers by including a large number of patients with NDBE progression, including samples from community-based practices, developing grading criteria designed to maximize specificity while preserving sensitivity, and including a prospective validation cohort. Given the results reported previously and our findings, there is clear evidence to support p53 IHC use as an adjuvant to routine histologic analysis.

## Supplementary Material

Supplementary Matierial

## Figures and Tables

**Table 1.** Clinical Characteristics of Patients in Retrospective Testing Cohorts

	NDBE at baseline		BE-IND at baseline		BE-LGD at baseline	
Characteristic	Nonprogressor	Progressor	Nonprogressor	Progressor	Nonprogressor	Progressor
Total	179	179 (38 EAC)	26	30 (3 EAC)	44	103 (16 EAC)

Age, *y*						
Median	67	66	65	62	68	66
Mean	66.0	65.7	67.3	63.6	68.4	65.7
Range	38–92	41–87	47–80	45–84	43–82	40–85

Sex, n (%)						
Female	25 (14.0)	25 (14.0)^a^	6 (23.1)	1 (3.3)^b^	14 (31.8)	16 (15.5)^b^
Male	154 (86.0)	154 (86.0)	20 (76.9)	29 (96.7)	30 (68.2)	87 (84.5)

Follow-up, *y*^c^						
Median	5.37	2.26	4.55	1.04	4.81	0.9
Mean	5.78	2.98	5.30	1.54	5.28	1.47
Range	3.07–13.68	0.09–9.9	3.29–9.02	0.21–4.72	3.03–11.14	0.06–11.06

Mean baseline to next endoscopy	3.11	1.96	2.05	0.78	1.31	0.85

Total accessions per patient						
Mean	3.13	2.73	3.58	2.6	4.48	2.45
Median	3	2	3	2	4	2
Range	2–9	2–9	2–6	2–4	2–11	2–8

Baseline pathology blocks per patient						
Mean	1.23	1.51^d^	2.04	1.57	2.02	1.91
Median	1	1	1	1	1	1
Range	1–6	1–7	1–8	1–5	1–10	1–8

All follow-up time points blocks per endoscopy						
Mean	1.25	1.67^e^	1.82	1.58	1.92	2.84^d^
Median	1	1	1	1	1	2
Range	1–6	1–7	1–7	1–3	1–13	1–14

a NDBE at baseline patients were sex-matched.

b Fisher exact test, *P* < .05.

c Selection criteria for patients were such that follow-up was longer in nonprogressors than progressors.

d Unpaired *t* test, *P* < .01.

e Unpaired *t* test, *P* < .0001.

## WHAT YOU NEED TO KNOW

### BACKGROUND AND CONTEXT

Barrett’s esophagus is the premalignant lesion of esophageal adenocarcinoma but has a low rate of progression. Biomarkers that can identify patients at high and low risk of progression would greatly aid surveillance strategies.

### NEW FINDINGS

p53 immunohistochemistry identifies a high-risk population of nondysplastic Barrett’s esophagus with high specificity and a high-risk population of Barrett’s esophagus with low-grade dysplasia with a high sensitivity in multiple community-acquired clinical cohorts.

### LIMITATIONS

Although mimicking “real world” conditions, the cohorts from nonacademic centers lacked rigorous control over surveillance intervals, clinical patient characteristics, and number of biopsies.

### IMPACT

p53 immunohistochemistry can help identify patients with nondysplastic Barrett’s esophagus who may benefit from more intensive surveillance and help determine which patients with low-grade dysplasia should be endoscopically treated.

## Abbreviations used in this paper:

BE: Barrett’s esophagus
BE-HGD: Barrett’s esophagus with high-grade dysplasia
BE-IND: Barrett’s esophagus indefinite for dysplasia
BE-LGD: Barrett’s esophagus with low-grade dysplasia
CI: confidence interval
EAC: esophageal adenocarcinoma
HR: hazard ratio
IHC: immunohistochemical
NDBE: nondysplastic Barrett’s esophagus
OR: odds ratio
