# Supplementary material for: Abnormal TP53 Predicts Risk of Progression in Patients With Barrett’s Esophagus Regardless of a Diagnosis of Dysplasia
Source: Gastroenterology. Author manuscript; Available in PMC 2022 Aug 1. (PMC9341495; doi:10.1053/j.gastro.2021.10.038)
Supplement: Supplementary Matierial [file NIHMS1824623-supplement-Supplementary_Matierial.pdf]

Supplementary Appendix

Supplementary Methods pg 2
Supplementary Figure Legends pg 8
Supplementary Tables pg 9
Supplementary Figures pg 19

Supplementary Methods

Development of p53 IHC scoring criteria

While much has been published regarding abnormal p53 expression in cancers and dysplasia, much less is known about expression patterns in NDBE. To further delineate the spectrum of p53 IHC staining in non-dysplastic biopsies, and delineate criteria for abnormal staining, we performed p53 IHC in 18 NDBE biopsies from patients with no known dysplasia, and in 115 NDBE biopsies that were concurrent with high grade dysplasia. The percentage of nuclei with positive staining was scored on an intensity scale of 0-3, with 0+ representing no staining and 3+ representing very strong staining. A consistent pattern of crypt staining was seen in all crypts in NDBE biopsies from patients with no known dysplasia, and in most crypts in NDBE biopsies from patients with concurrent high-grade dysplasia: crypt base positivity was always present, with most cells having 0-1+ nuclear positivity, scattered cells having 2+ nuclear positivity, and rare cells having 3+ nuclear positivity (**Supplementary Figure S6**). This crypt base positivity diminished towards the surface, which typically had 0 to scattered 1+ IHC positivity. This staining pattern is consistent with the physiologic normal expression of p53 in the proliferative zone of the crypt base.

In addition to this normal p53 crypt expression pattern, NDBE biopsies from patients with concurrent high-grade dysplasia harbored foci of distinctly abnormal staining comprised of two common patterns: markedly increased staining, and complete absence of staining (**Supplementary Figure S6**). Increased p53 expression is known to be associated with *TP53* point mutations; these result in protein stabilization and nuclear accumulation.

Absent p53 expression is known to be associated with *TP53* truncation mutations, and other genomic aberrations that result in loss of p53 expression. To develop criteria for abnormally increased staining, we identified the foci that had the strongest staining, and scored the percentage of cells that had either 2+ and 3+ nuclear positivity. Increased staining extended onto the luminal surface in some cases, but was confined to the crypt base in others, in which case only the crypt base was evaluated. Foci as small as a single individual crypt base were considered adequate for scoring increased staining. Scoring using either 3+ positivity alone, or combined 2-3+ positivity, revealed that approximately 35% of NDBE biopsies from patients with concurrent high-grade dysplasia had foci of increased p53 expression that was greater than anything present in the NDBE biopsies without dysplasia (**Supplementary Figure S7**). We chose >50% 2-3+ nuclear positivity as a cut-off to define abnormal p53 expression because it had 100% specificity for an association with concurrent high-grade dysplasia, and could be rapidly assessed by a pathologist, potentially making it a practical biomarker for routine clinical use. Finally, occasional NDBE biopsies from patients with concurrent high-grade dysplasia also had focal marked p53 positivity present only within surface epithelium, but not adjacent crypts. This finding was consistent with a mutant p53 clone extending onto the surface from a nearby crypt not present in the section. Therefore, the presence of 2-3+ p53 positivity in >50% of a contiguous focus of 20 surface cells was also considered abnormal.

While the vast majority of crypts from NDBE biopsies with no known dysplasia had ≤20% 2-3+ nuclear positivity in crypt bases, there were some crypts with 31-40% 2-3+ nuclear positivity. Although this degree of positivity was present at higher frequency in NDBE

biopsies from patients with concurrent high-grade dysplasia, there was appreciable overlap. Therefore, 2-3+ nuclear positivity in 21-50% of crypt epithelial nuclei was considered equivocal, and while underlying *TP53* mutations may be present in a subset of these cases, was classified as p53-NL in all analyses in this study.

The second common abnormal p53 expression pattern, complete absence of p53 expression, was identified in 20/115 (17%) NDBE biopsies from patients with concurrent high-grade dysplasia compared to none of the NDBE biopsies from patients with no known dysplasia. Because of the consistent normal p53 expression pattern present in crypt bases of NDBE biopsies from patients with no known dysplasia, this abnormal loss of staining could be reliably scored when identified in foci as small as a single crypt base. In contrast to abnormally increased p53 expression, the very low (normal) p53 expression in Barrett's epithelium at the luminal surface makes it impossible to accurately diagnose absent p53 expression (mutation) when the involvement is limited to the surface epithelium of a NDBE biopsy.

Finally, during the blinded slide review of the retrospective cohort, we identified one additional distinctive rare abnormal p53 expression pattern: complete absence of nuclear p53 positivity in the presence of strong cytoplasmic positivity. This abnormal pattern has been seen for some other nuclear proteins as well as in p53 and has been attributed to mutations that result in loss of the nuclear localization domain[39]. This abnormal pattern was considered to be a variant of loss of expression and was combined with absent

staining pattern in subsequent analyses. **Supplementary Table S1** summarizes the p53 expression criteria used in this study.

The criteria defined above were further validated in an additional sample set of 50 NDBE biopsies from patients with no known dysplasia, and 50 BE-HGD biopsies. Abnormal p53 expression was found in 2/50 (4%) of the NDBE biopsies and 48/50 (96%) BE-HGD biopsies, providing additional validation that these scoring criteria are very sensitive for identification of abnormal p53 in advanced Barrett's neoplasms, while only being positive in a very small subset of unselected NDBE biopsies (which is to be expected for a possible biomarker).

Central Pathology Review

When there were discrepancies between the original diagnosis and reviewed diagnosis, a third independent reviewer assessed the slides. Any remaining discrepancies were assessed by consensus at a multi-headed microscope. Primary analysis was performed using the central review pathologic diagnosis. A secondary analysis using the original pathologic diagnosis was also performed to validate the utility of p53 IHC in the context of the original routine clinical diagnosis. For the prospective cohort, to determine how the p53 IHC protocol would work in routine practice, we utilized the original clinical diagnosis except all diagnoses of dysplasia or cancer were confirmed by at least one additional pathologist.

For the retrospective testing cohorts, central pathology review confirmed the presence of Barrett's mucosa, columnar dysplasia or adenocarcinoma in 3243 biopsies. IHC for p53 was not attempted in 353 of these cases because there was no tissue available, or the histologic focus of interest was not present in remaining tissue. Of the remaining 2890 biopsies, a p53 IHC result was obtained in 2853 (98.7%). **Figure 1b** shows examples of histologic findings and p53 IHC staining from both non-progressors and progressors, across the complete spectrum of precursor diagnoses in this study.

For the prospective validation cohort, to determine how frequent p53 IHC staining was performed, a random sampling of 500 cases of BE-IND and BE-LGD each revealed that p53 IHC was performed on 68.2% and 59.4% respectively. For NDBE, p53 IHC was utilized exclusively by a group of 7 pathologists and was performed in 57.2% of cases. IHC for p53 was performed on all lesional blocks in a given case after histopathologic review by a pathologist. To further evaluate for bias based upon reading pathologists, we calculated the rate of p53-ABNL at the individual locations for BE-LGD.

**P53**	**Needham, MA**	**Irving, TX**	**Phoenix, AZ**
Normal	27	72	23
Point mutant	71	146	32
Absent	9	22	1
Both point and absent	4	4	1
**Total ABNL Freq**	**84/111 = 75.7% (95% CI 66.4-83.1)**	**172/244 = 70.5% (95% CI 64.3-76.0)**	**32/57 = 56.1% (95% CI 42.4-69.0)**

While there was a slight difference in the rate of called p53-ABNL between the center with the fewest numbers and the other two centers (see chart), given the 95% CIs the difference was relatively minor and unlikely to have major bearing on the results of the study or the applicability for clinical use. It is unknown if this difference is due to a systemic

difference in the patient population at the different locations, differences in diagnosing BE-LGD, or differences in p53 IHC. Regardless, overall across a multi-year time span and many different pathologist we were able to identify a p53-ABNL population at higher risk of progression.

TP53 sequencing

In samples that had at least one remaining unstained slide, areas of BE were macrodissected for DNA isolation and sequencing of all exons of the *TP53* gene. Library construction was performed on any sample with at least 2ng of total DNA using the Pillar Biosciences (Natick, MA) SLIMAmp target enrichment technology, whereby all exons of *TP53* were amplified with multiplexed PCR and the resulting material utilized for next-generation sequencing. Samples were pooled and sequenced on an Illumina Miseq. The Pillar PIVAT analysis software was used to analyze the results.

To determine likely somatic, pathogenic *TP53* mutations, as series of filtering steps were performed. All mutational calls under 0.025 allele frequency, present in any ethnic background at > 0.001 in the gnomAD database (https://gnomad.broadinstitute.org/about), or were not within the coding region or splice region were discarded. Additionally, missense mutations that have not been previously reported in esophageal adenocarcinoma (cbioPortal) or were not reported in the COSMIC database at least 5 times were removed. Predicted nonsense, frameshift, or splice altering mutations were retained.

Supplementary Figure Legends

Supplementary Figure S1. Cartoon of endoscopy and biopsy terminology. Diagram illustrates determination of baseline or later surveillance endoscopies as positive (p53-ABNL) or negative (p53-NL) by biopsies. When looking at the endoscopy level, if any biopsy/pathology block at a given endoscopy is p53-ABNL the endoscopy is considered to be positive/abnormal.

Supplementary Figure S2. p53-ABNL vs time to progression. The percentage of p53-ABNL endoscopies for progression patients was plotted as a function of time for each histologic diagnosis.

Supplementary Figure S3. Timeline charts of subjects with intramucosal (A) and invasive carcinoma (B). Multiple patients had progression from NDBE to invasive cancer within the recommended surveillance interval without a diagnosis of dysplasia. The timing of the different endoscopies, p53 status, and histologic diagnosis are plotted.

Supplementary Figure S4. Kaplan-Meier analysis of progression free survival in patients with screening or surveillance baseline endoscopies. Kaplan-Meier analysis for progression free survival split between patients with a true index endoscopy as baseline or a surveillance endoscopy for NDBE or LGD as baseline. Baseline is defined as the first endoscopy with a diagnosis of Barrett's esophagus seen at Inform Diagnostics. Progression from NDBE or BE-IND to LGD, HGD, or EAC (top) and NDBE, BE-IND, or LGD to HGD or EAC (bottom).

Supplementary Figure S5. Kaplan-Meier analysis of progression free survival in patients with screening or surveillance baseline endoscopies stratified by p53 IHC status. Kaplan-Meier curves for progression to either BE-LGD/BE-HGD/EAC (left side) or to BE-HGD/EAC (right side) free survival in patients with p53-ABNL and p53-NL. All patient numbers and statistical analysis are reported in supplementary table S10. * denotes $p<0.05$ for p53-NL vs p53-ABNL.

Supplementary Figure S6. p53 IHC scoring A) example of cells negative p53 IHC (blue arrow), 1+ staining (tan arrow), 2+ staining (light brown arrow), and 3+ staining (dark brown arrow). B) example of wild type staining. C) Example of positive nuclear staining (p53-ABNL). D) Example of absent staining (p53-ABNL). Glands quantified in Blue circles.

Supplementary Figure S7. Distribution of p53 IHC positivity in NDBE biopsies. Bar chart showing numbers of samples with grading of 0, 1+, 2+, 3+ p53 IHC in NDBE biopsies.

Supplementary Figure 8. Surveillance schema for p53-ABNL and p53-NL Barrett's esophagus. For patients with BE-LGD or BE-IND (after optimized acid suppression), a p53-ABNL results suggests a higher rate of progression and thus endoscopic therapy would be recommended. For p53-NL BE-LGD or BE-IND, risk of progression is lower and continued surveillance is likely most appropriate. Patients with p53-ABNL NDBE have a rate of progression similar to BE-LGD and thus may benefit from yearly surveillance as long as biopsies remain p53-ABNL. After two consecutive endoscopies showing p53-NL NDBE, patients are likely at low risk of progression and could have their surveillance interval lengthened to 5 years.

SUPPLEMENTARY TABLES

Supplementary Table S1. p53 immunohistochemistry scoring criteria

**p53 Expression Pattern**	**Criteria**	**Categorization for Analyses**
Normal (wild-type) pattern	2-3+ nuclear positivity in $\leq$20% of epithelial cells in all individual crypts or glandular profiles, and all contiguous stretches of surface epithelium	p53-NL
Equivocal pattern	2-3+ nuclear positivity in 21-50% of cells in at least one crypt base or glandular profile, or within a contiguous focus of at least 20 surface epithelial cells	p53-NL
Point mutation pattern	2-3+ nuclear positivity in >50% of cells in at least one crypt base or glandular profile, or within a contiguous focus of at least 20 surface epithelial cells	p53-ABNL
Absent (null) pattern	Total absence of staining in all epithelial cells of at least one crypt base or glandular profile*	p53-ABNL
Cytoplasmic pattern	Absence of nuclear staining with aberrant cytoplasmic staining in all epithelial cells of at least one crypt base or glandular profile*	p53-ABNL

*Diagnosing the absent or cytoplasmic patterns required the presence of internal control positivity (nuclear positivity in crypt epithelium, squamous epithelium, immune cells or stromal cells) as evidence of stain adequacy.

Supplementary Table S2. Study subject eligibility and inclusion

	Eligible	Excluded (%)[1]	Included same class (%)	Included Re-classified (%)[2]	Final Total Subjects (with re-classified)
Total subjects	672	111 (16.5%)	515 (76.6%)	46 (6.8%)	561
Non-progressors	313	63 (20.1%)	241 (77.0%)	9 (2.9%)	249
NDBE	195	16 (8.2%)	179 (91.8%)	0	179
BE-IND	53	27 (50.9%)	22 (41.5%)	4 (7.5%)	26
BE-LGD	65	20 (30.8%)	40 (61.5%)	5 (7.7%)	44
Progressors	359	48 (13.4%)	274 (76.3%)	37 (10.3%)	312
NDBE	196	10 (5.1%)	170 (86.7%)	16 (8.2%)	179
BE-IND	45	9 (20%)	22 (48.9%)	14 (31.1%)	30
BE-LGD	118	29 (24.6%)	82 (69.5%)	7 (5.9%)	103

[1]See Supplementary Table 1 for details of excluded cases.

[2]Most re-classifications were due to a central path review change of the baseline diagnosis; see Supplementary Table 4 for details.

Supplementary Table S3. Details of excluded study subjects

Category	Total	Details
Non-progressors	63	
NDBE	16	Baseline not available: 15
		Final follow-up biopsy upgraded to BE-IND: 1
BE-IND	27	Baseline downgraded to NDBE: 22
		Baseline not available: 4
		Final follow-up upgraded to BE-LGD: 1
BE-LGD	20	Baseline downgraded to NDBE: 14
		Baseline not available: 5
		Baseline upgraded to BE-HGD: 1
Progressors	48	
NDBE	10	Baseline not available: 6
		Progression not confirmed: 4
BE-IND	9	Baseline not available: 4
		Baseline upgraded to BE-HGD: 4
		Progression not confirmed: 1
BE-LGD	29	Baseline upgraded to BE-HGD: 19
		Baseline NA: 9
		Progression not confirmed: 2

Supplementary Table S4. Details of re-classified study subjects

Category		Total	Details
Non-progressor to non-progressor		8	
	BE-IND to BE-LGD	4	Baseline upgraded from BE-IND to BE-LGD
	BE-LGD to BE-IND	4	Baseline downgraded from BE-LGD to BE-IND
Non-progressor to progressor			
	BE-LGD to NDBE	1	Final follow-up upgraded from BE-LDG to BE-HGD; NDBE prior to first BE-LGD available for new baseline
Progressor to progressor		37	
	NDBE to BE-IND	4	Baseline upgraded from NDBE to BE-IND: 3<br>Baseline not available: 1
	NDBE to BE-LGD	12	Baseline upgraded from NDBE to BE-LGD: 8<br>Baseline not available: 4
	BE-IND to NDBE	5	Baseline downgraded from BE-IND to NDBE: 5
	BE-IND to BE-LGD	9	Baseline upgraded from BE-IND to BE-LGD: 7<br>Baseline not available: 2
	BE-IND to NDBE	3	Baseline downgraded from BE-IND to NDBE: 3
	BE-LGD to BE-IND	4	Baseline downgraded from BE-LGD to BE-IND: 4
Progressor to non-progressor		0	Progression not confirmed: 7, all excluded from study

Supplementary Table S5. p53 IHC vs age

	Total	Youngest age quartile	Lower middle age quartile	Upper middle age quartile	Oldest age quartile	
All non-progressors						
NDBE	943	9/236 (3.8%)	13/236 (5.5%)	21/236 (8.9%)	15/235 (6.4%)	NS
BE-IND	63	2/16 (12.5%)	3/16 (18.7%)	2/16 (12.5%)	2/15 (13.3%)	NS
BE-LGD	121	14/31 (46.7%)	10/30 (33.3%)	12/30 (40.0%)	5/30 (16.7%)	P<0.05 for oldest quartile vs all others
All Progressors						
NDBE	620	68/155 (43.9%)	62/155 (40.0%)	73/155 (47.1%)	66/155 (42.6%)	NS
BE-IND	76	16/19 (84.2%)	17/19 (89.5%)	17/19 (89.5%)	19/19 (100%)	NS
BE-LGD	298	69/75 (92.0%)	68/75 (90.7%)	73/74 (98.6%)	72/74 (97.3%)	NS

Supplementary Table S6. p53 IHC abnormalities in baseline endoscopies summarized for each subject[1]

Diagnostic category at baseline	Progression Status	Total patients with specified baseline diagnosis	Number of specified baseline endoscopies with abnormal p53 IHC	Percent	Chi square P-value[1]
NDBE	Non-progressor	179	3	1.7%	
	Progressor	179	91	50.8%	<0.00001
BE-IND	Non-progressor	26	4	15.4%	
	Progressor	30	27	90.0%	<0.00001
BE-LGD	Non-progressor	44	20	45.4%	
	Progressor	103	97	94.2%	<0.00001

[1]The results of baseline biopsies in individual patients were summarized as follows: if there were multiple biopsies with Barrett's mucosa negative for dysplasia, the subject was considered to have abnormal p53 IHC if any one biopsy was abnormal.

[2]Non-progressor vs progressor for each diagnostic category

Supplementary Table S7. p53 IHC abnormalities at baseline with each block/biopsy counted separately

Diagnostic category at baseline	Progression Status	Total pathology blocks with specified baseline diagnosis	Number of specified baseline pathology blocks with abnormal p53 IHC	Percent	Chi square P-value[1]
NDBE	Non-progressor	219	3	1.4%	
	Progressor	279	112	40.1%	<0.00001
BE-IND	Non-progressor	29	3	10.3%	
	Progressor	32	27	84.8%	<0.00001
BE-LGD	Non-progressor	55	20	36.4%	
	Progressor	131	122	93.1%	<0.00001

[1]Non-progressor vs progressor for each diagnostic category

Supplementary Table S8. p53 IHC abnormalities in baseline biopsies/pathology blocks using original final diagnosis (not central pathology review results)

Diagnostic category at baseline	Total biopsies with specified baseline diagnosis	Number of specified baseline biopsies with abnormal p53 IHC	Percent	Chi square P-value[1]
Non-progressor				
Negative for dysplasia	194	3	1.5%	
Indefinite for dysplasia	59	10	15.1%	
Low grade dysplasia	71	26	36.6%	
Progressor				
Negative for dysplasia	259	106	40.9%	<0.00001
Indefinite for dysplasia	38	26	68.4%	<0.00001
Low grade dysplasia	139	129	92.8%	<0.00001

[1]Non-progressor vs progressor

Supplementary Table S9. p53 IHC abnormalities in baseline endoscopies summarized for each subject using original final diagnosis (not central pathology review results)

Diagnostic category at baseline	Total subjects in baseline diagnostic category	Number of subjects with abnormal p53 IHC in any baseline biopsy	Percent	Chi square P-value[1]
Non-progressor				
Negative for dysplasia	179	3	1.7%	
Indefinite for dysplasia	27	5	18.5%	
Low grade dysplasia	38	18	47.4%	
Progressor				
Negative for dysplasia	179	89	51.1%	<0.00001
Indefinite for dysplasia	33	24	72.7%	<0.0001
Low grade dysplasia	96	90	93.7%	<0.00001

[1]Non-progressor vs progressor

Table S10. p53 IHC abnormalities in Prospectively tested validation cohort separated by index and surveillance endoscopies

Patient Category	Diagnosis of Index p53 Biopsy	p53 IHC Normal	p53 IHC Abnormal	Progression Free Survival Endpoint	Log Rank Test	P-Value
p53 IHC on first Barrett's biopsy (Index)						
	NDBE	260	10	LGD/HGD/EAC	Z=6.52	P<0.001
				HGD/EAC	Z=2.81	P=0.0049
	BE-IND	97	41	LGD/HGD/EAC	Z=3.99	P<0.001
				HGD/EAC	Z=2.09	P=0.037
	BE-LGD	30	79	HGD/EAC	Z=1.97	P=0.049
p53 IHC on known Barrett's surveillance biopsy (no prior BE-IND or dysplasia)						
	NDBE	356	20	LGD/HGD/EAC	Z=10.07	P<0.001
				HGD/EAC	Z=2.65	P=0.0081
	BE-IND	161	90	LGD/HGD/EAC	Z=2.98	P=0.0029
				HGD/EAC	Z=2.19	P=0.0014
	BE-LGD	65	174	HGD/EAC	Z=2.61	P=0.0092
p53 IHC on known BE-LGD patient						
	BE-LGD	26	30	HGD/EAC	Z=2.08	P=0.038

SUPPLEMENTARY FIGURES

Supplementary Figure S1. Cartoon of endoscopy and biopsy terminology.

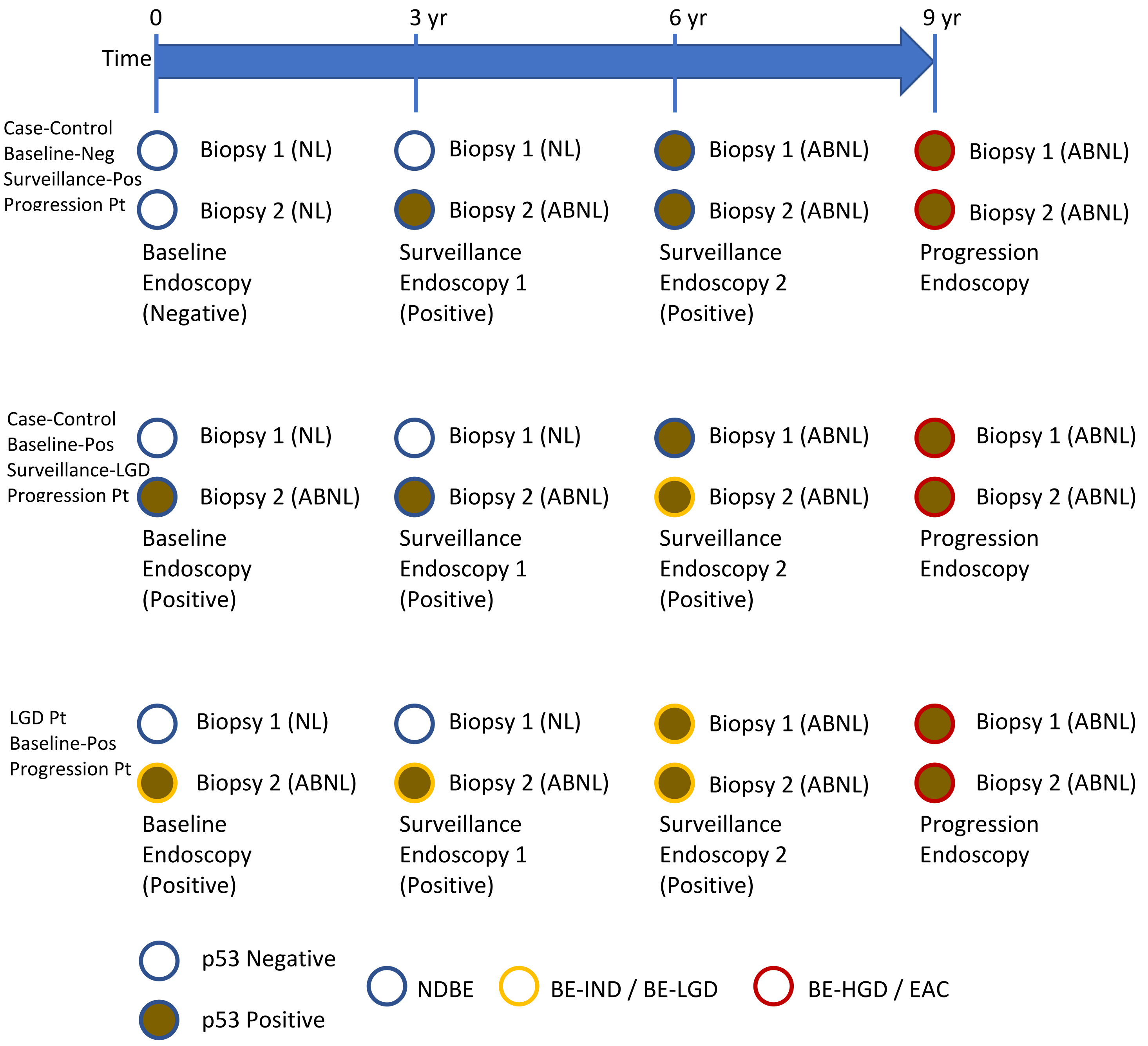

Supplementary Figure S2. p53-ABNL vs time to progression.

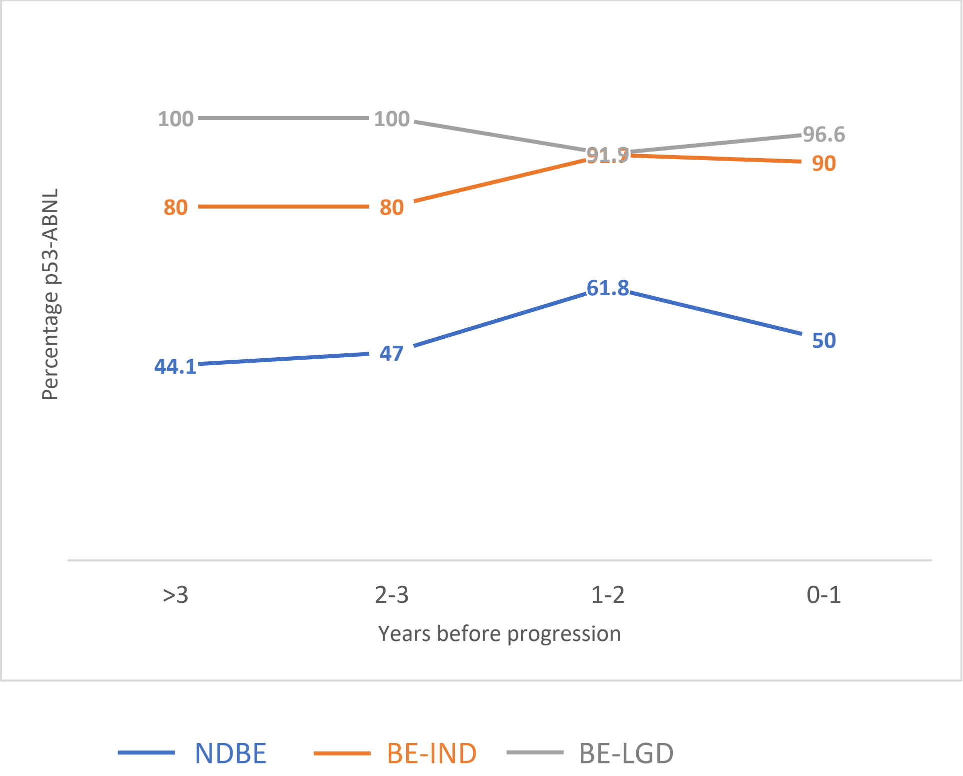

Supplementary Figure S3. Timeline charts of subjects with intramucosal (A) and invasive carcinoma (B). Multiple patients had progression from NDBE to invasive cancer within the recommended surveillance interval without a diagnosis of dysplasia.

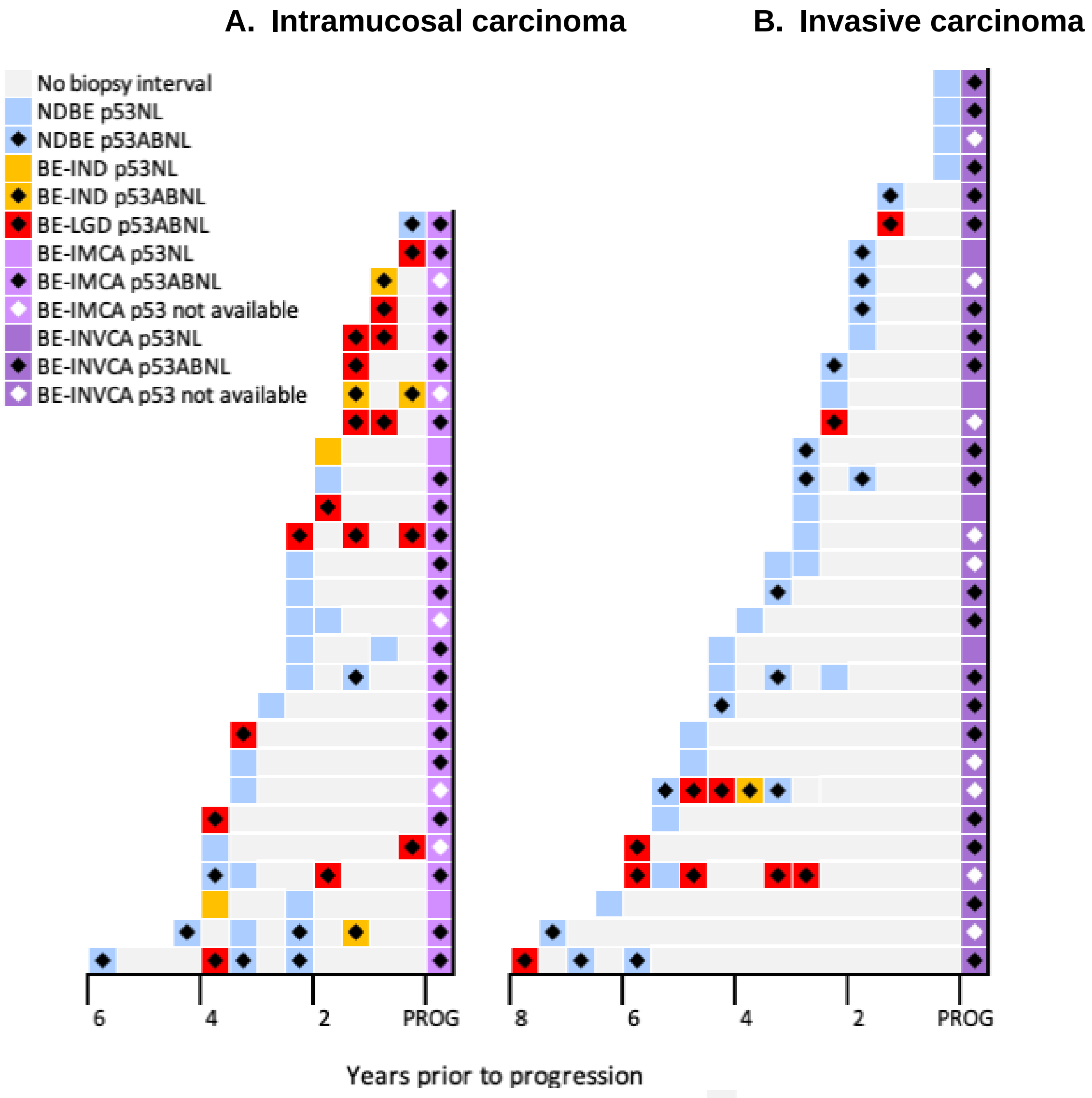

Supplementary Figure S4. Kaplan-Meier analysis of progression free survival in patients with screening or surveillance baseline endoscopies.

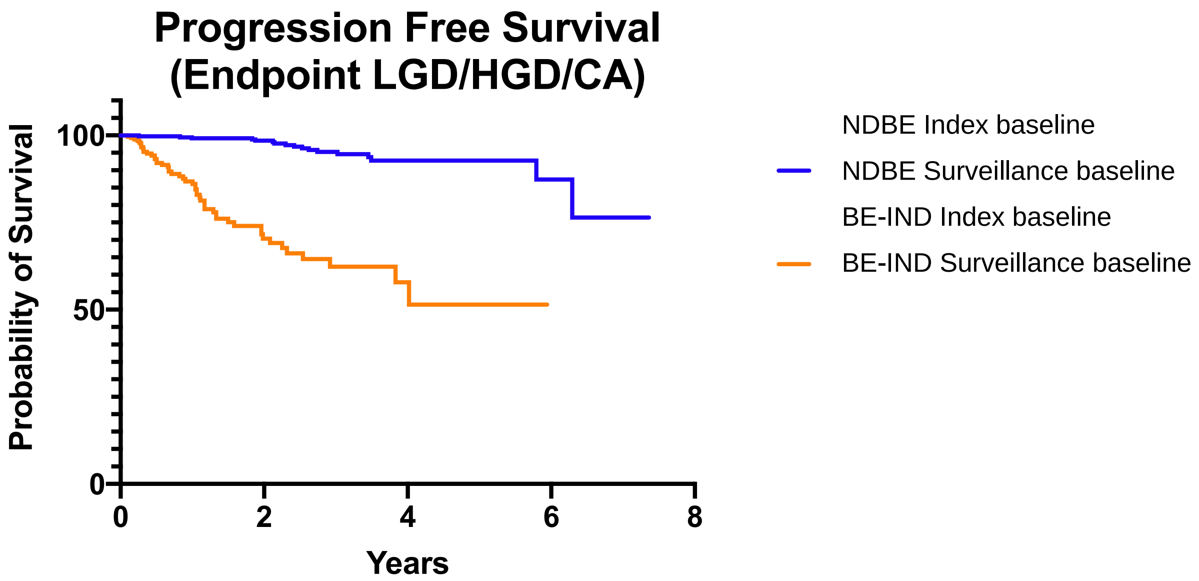

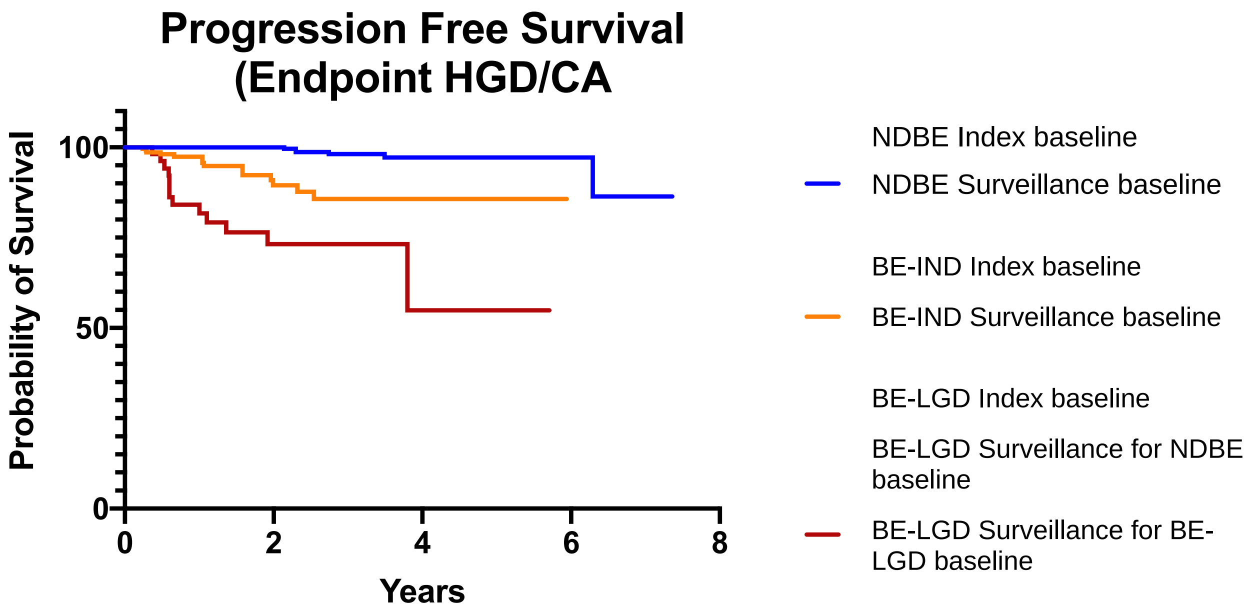

Supplementary Figure S5. Kaplan-Meier analysis of progression free survival in patients with screening or surveillance baseline endoscopies stratified by p53 IHC status. Kaplan-Meier curves for progression to either BE-LGD/BE-HGD/EAC (left side) or to BE-HGD/EAC (right side) free survival in patients with p53-ABNL and p53-NL. All patient numbers and statistical analysis are reported in supplementary table S10. * denotes p<0.05 for p53-NL vs p53-ABNL.

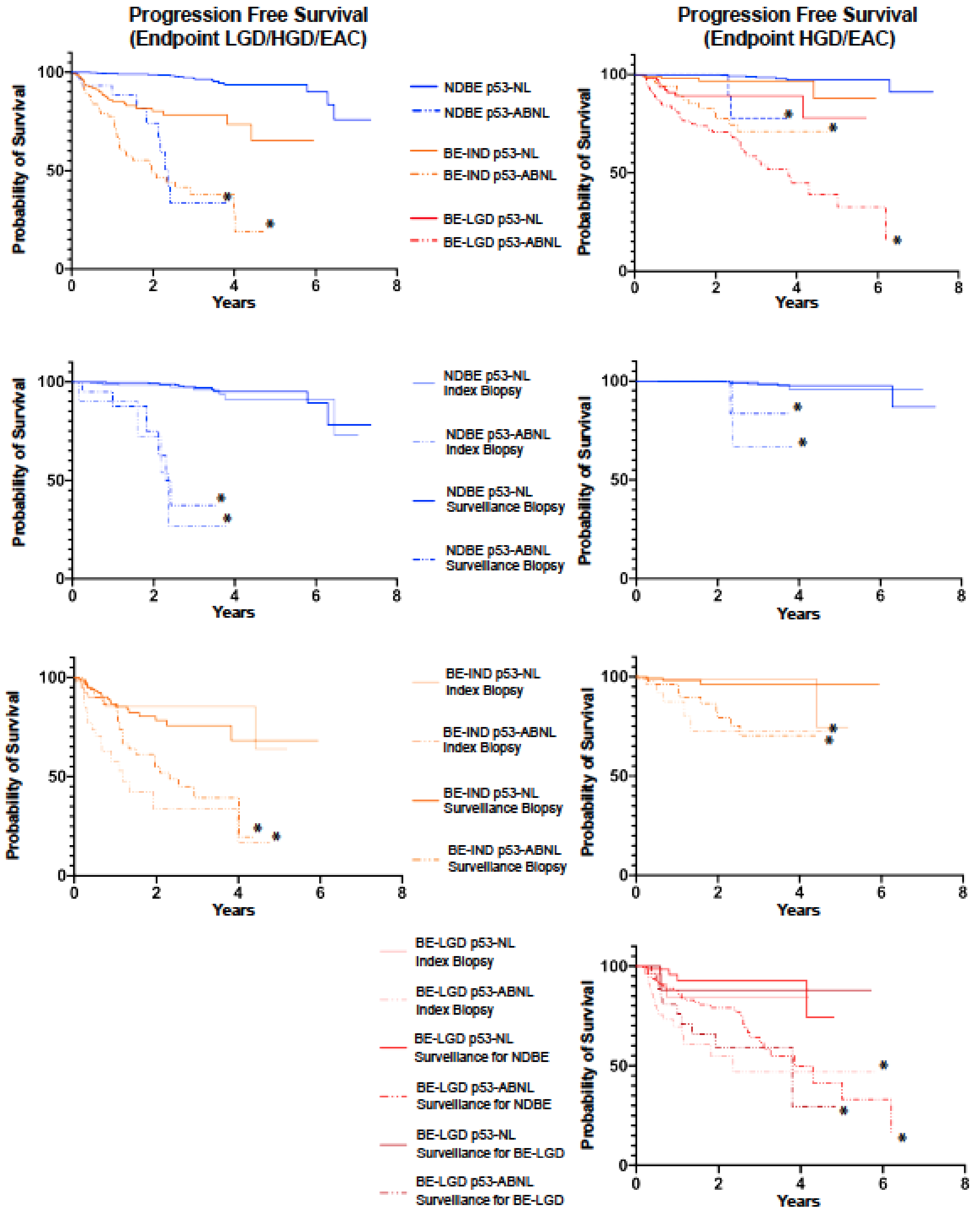

Supplementary Figure S6. p53 IHC scoring A) example of cells with negative p53 IHC (blue arrow), 1+ staining (tan arrow), 2+ staining (light brown arrow), and 3+ staining (dark brown arrow). B) example of wild type staining. C) Example of positive nuclear staining. D) Example of absent staining (positive). Glands quantified in Blue circles.

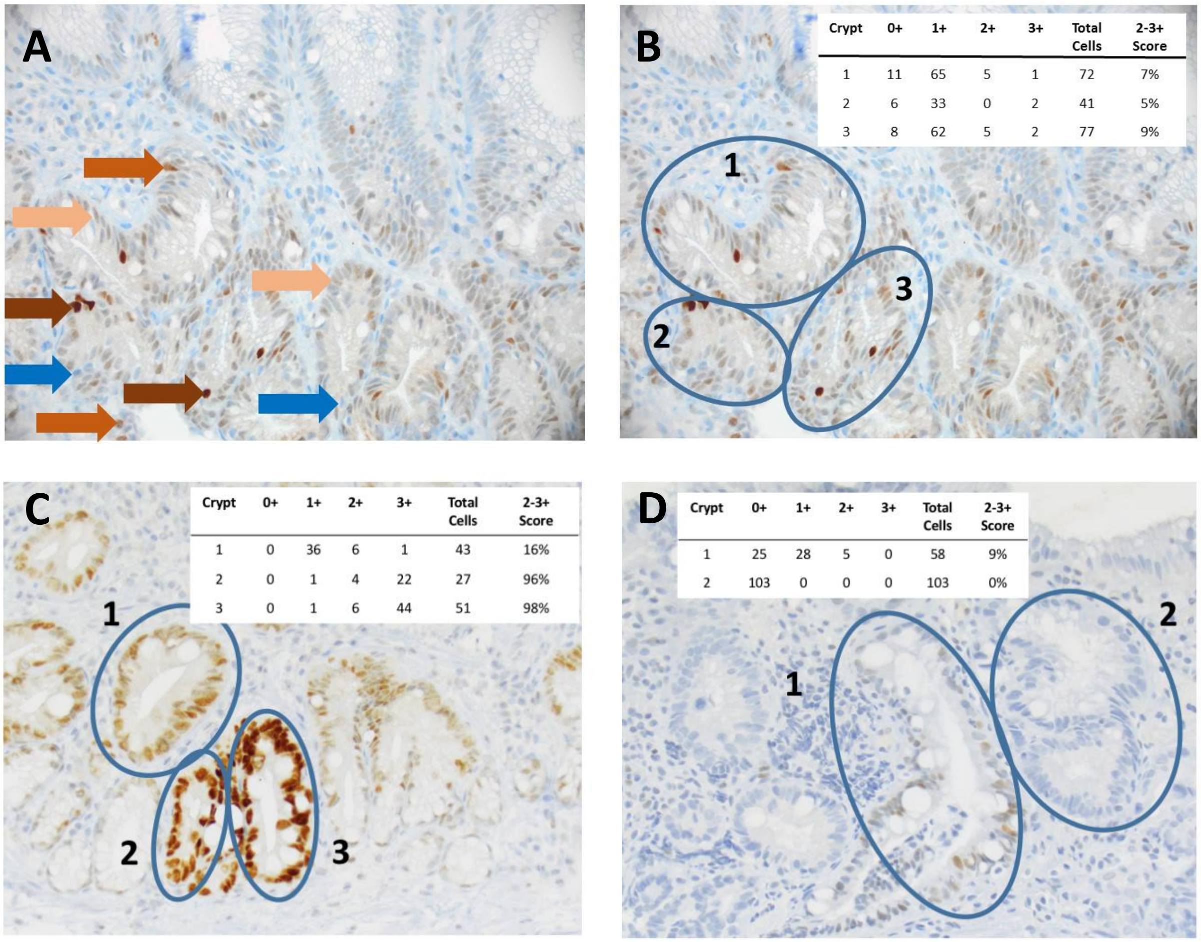

Supplementary Figure S7. Distribution of p53 IHC positivity in NDBE biopsies

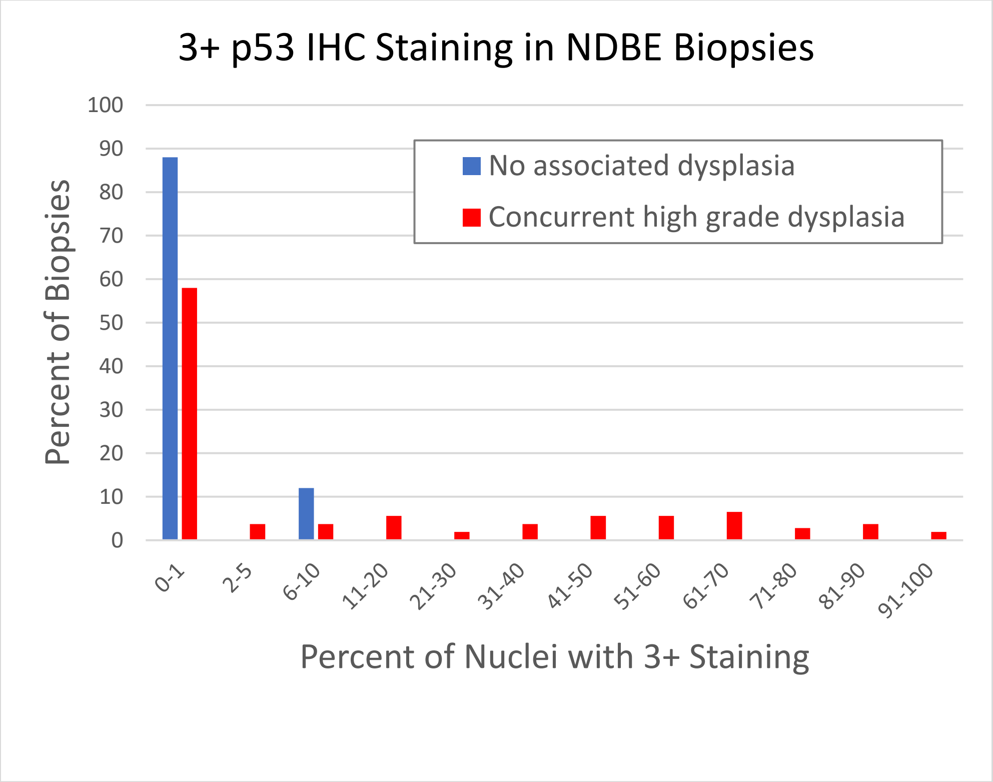

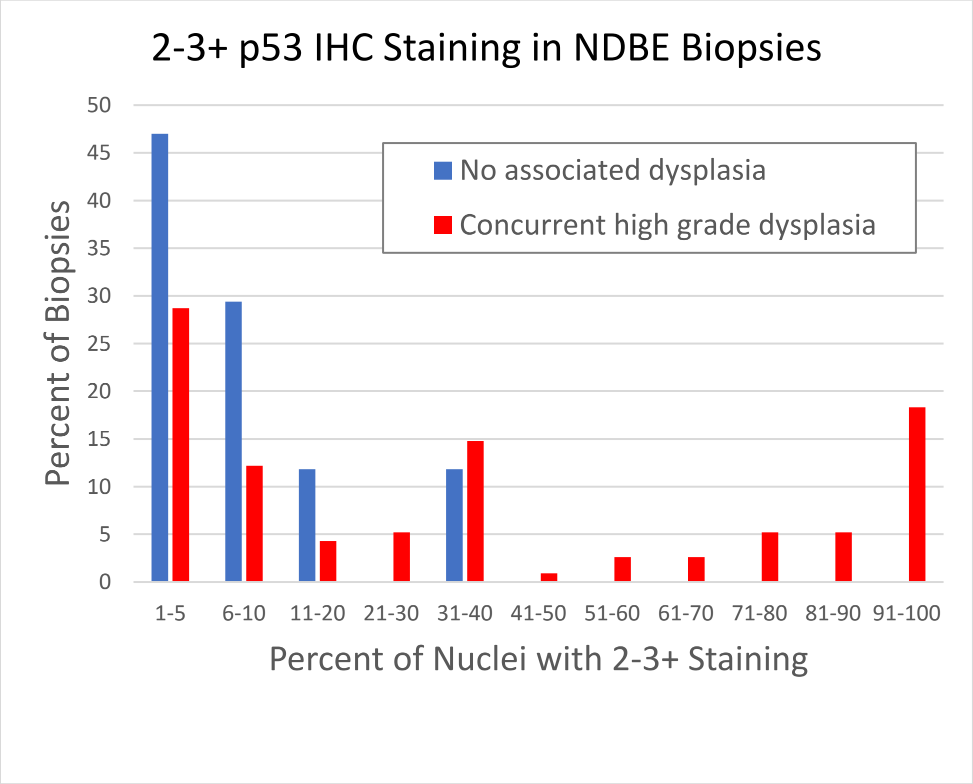

Supplementary Figure 8. Surveillance schema for p53-ABNL and p53-NL Barrett's esophagus

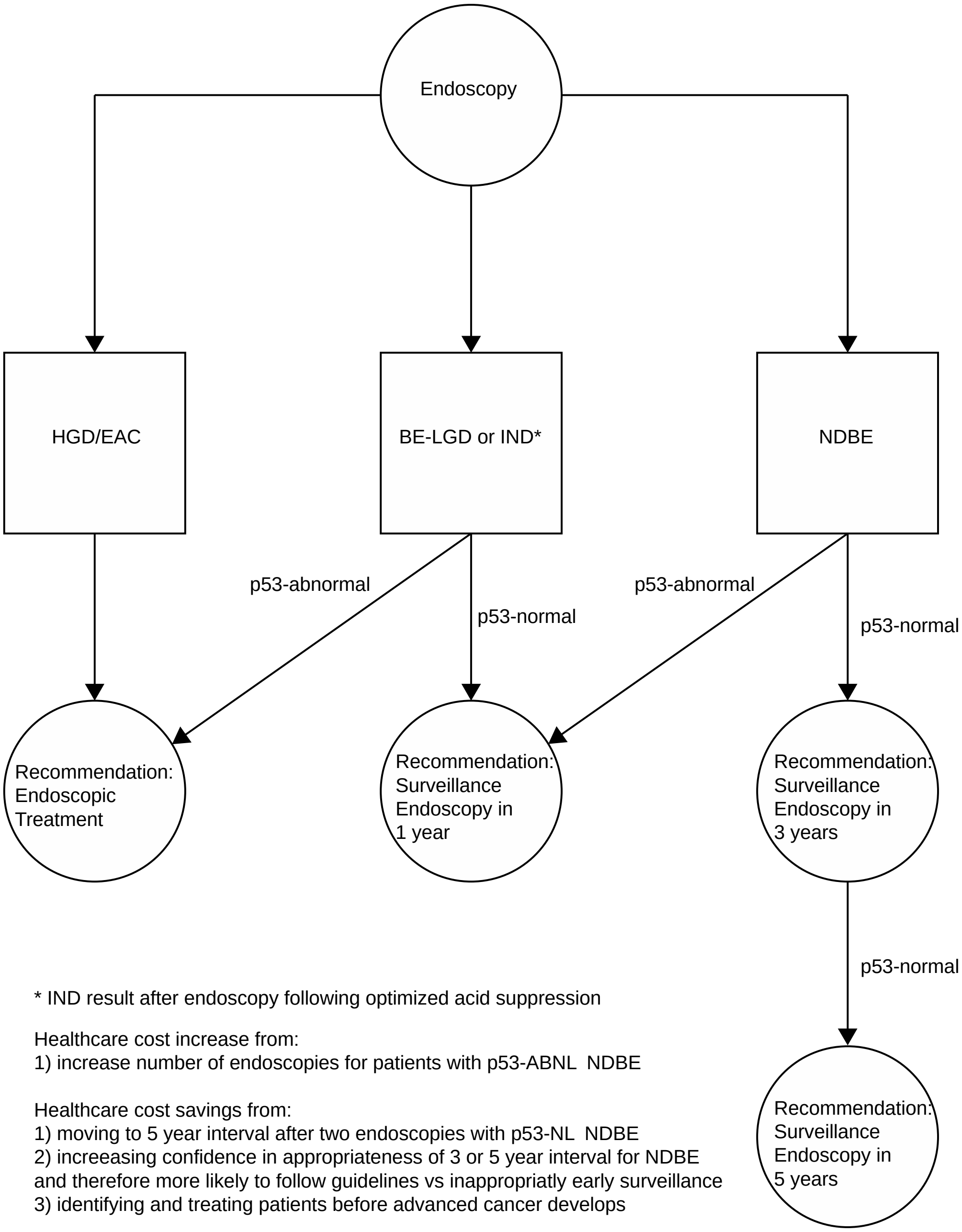

* IND result after endoscopy following optimized acid suppression

Healthcare cost increase from:
1) increase number of endoscopies for patients with p53-ABNL NDBE

Healthcare cost savings from:
1) moving to 5 year interval after two endoscopies with p53-NL NDBE
2) increeasing confidence in appropriateness of 3 or 5 year interval for NDBE and therefore more likely to follow guidelines vs inappropriatly early surveillance
3) identifying and treating patients before advanced cancer develops
